# The cGAS-STING pathway in senescence and aging-related diseases: mechanisms and therapeutic opportunities

**DOI:** 10.1186/s12964-026-02855-7

**Published:** 2026-04-01

**Authors:** Shiqing Tan, Eugenie Nepovimova, Marian Valko, Klaudia Jomova, Qinghua Wu, Kamil Kuca

**Affiliations:** 1 College of Life Science, Yangtze University, Jingzhou, 434025 China; 2 Department of Chemistry, Faculty of Science, University of Hradec Králové, 500 03 Hradec Králové, Czech Republic; 3 Center of Advanced Innovation Technologies, VSB-Technical University of Ostrava, 70800 Ostrava-Poruba, Czech Republic; 4 Faculty of Chemical and Food Technology, Slovak University of Technology, 812 37 Bratislava, Slovakia; 5 Department of Chemistry, Faculty of Natural Sciences, Constantine the Philosopher University in Nitra, Nitra, 949 74 Slovakia; 6 Faculty of Informatics and Management, University of Hradec Kralove, 500 03, Hradec Kralove, Czech Republic; 7 Biomedical Research Center, University Hospital Hradec Kralove, 50005 Hradec Kralove, Czech Republic

**Keywords:** cGAS-STING, Cellular senescence, Genomic instability, Mitochondrial dysfunction, Aging-related diseases

## Abstract

The cGAS-STING pathway acts as a critical molecular hub connecting genomic instability with cellular senescence. Functioning as a key regulator of the innate immune system, this pathway detects aberrant cytoplasmic DNA to activate downstream inflammatory responses, thereby playing a pivotal role in aging-related diseases. This review systematically explores the core mechanisms by which the cGAS-STING pathway regulates cellular senescence, emphasizing its role in triggering senescence through the recognition of DNA damage signals (e.g., oxidative stress, telomere dysfunction) and promoting paracrine senescence effects via the production and release of the senescence-associated secretory phenotype (SASP). We focus on the crosstalk between this pathway and current research hotspots, including the hypoxic microenvironment, ammonia-induced cell death, neutrophil extracellular trap (NET) formation, and macrophage polarization, uncovering its intricate molecular network in cellular senescence regulation. Moreover, this review provides an in-depth analysis of the cGAS-STING pathway's pathological contributions and molecular mechanisms in aging-related diseases, along with a summary of potential therapeutic strategies targeting this pathway, based on recent advances. These findings provide a critical theoretical framework for understanding cellular senescence mechanisms and advancing anti-aging interventions.

## Introduction

Aging constitutes a progressive physiological decline that intrinsically underpins the pathogenesis of numerous chronic diseases [1, 2]. Central to this process is inflammaging, a state of persistent, low-grade systemic inflammation recognized as a critical pathological driver [3, 4]. Among the established hallmarks of aging, cellular senescence has emerged as a focal point of research due to its capacity to disrupt tissue homeostasis and propel disease progression [5–7]. Senescent cells are defined by an irreversible cell cycle arrest, accrual of macromolecular damage, and most notably, the development of a potent senescence-associated secretory phenotype (SASP) [8–10]. Through the chronic secretion of pro-inflammatory cytokines, chemokines, and proteases, the SASP fosters a deleterious microenvironment that perpetuates tissue dysfunction and accelerates the aging trajectory [11, 12]. Standing at the nexus of these phenomena is the cGAS-STING (cyclic GMP-AMP synthase–stimulator of interferon genes) innate immune pathway, which functions as a principal molecular conduit integrating genomic instability with chronic inflammation and cellular senescence [13].

Acting as a cytosolic DNA sensor, cGAS responds to aberrant double-stranded DNA (dsDNA) by catalyzing the synthesis of the second messenger cyclic GMP-AMP (cGAMP) [14]. This ligand subsequently activates STING, initiating downstream signaling cascades—primarily the TANK-binding kinase 1 (TBK1)-interferon regulatory factor 3 (IRF3) and nuclear factor kappa B (NF-κB) axes—that culminate in the production of type I interferons (IFN-Is) and key SASP components [14, 15]. Pathological hyperactivation of this pathway is implicated not only in the cell-autonomous initiation of senescence but also in its propagation via SASP-mediated paracrine effects on neighboring bystander cells [16–18]. Furthermore, emerging research delineates a complex interplay between cGAS-STING and other senescence-associated processes, including hypoxic stress, neutrophil extracellular trap (NET) formation, and macrophage polarization, suggesting a collaborative role in modulating the senescent landscape [19–21]. In contexts such as ammonia-induced cell death, mitochondrial dysfunction and concomitant oxidative stress are posited to coregulate cGAS-STING signaling, although the precise mechanistic networks remain to be fully delineated [22, 23]. Critically, dysregulated cGAS-STING activation is increasingly implicated in the pathophysiology of a spectrum of aging-related conditions—encompassing cardiovascular, metabolic, musculoskeletal, and ophthalmic diseases—where it acts as a significant driver of pathology [24–27].

Given its central role as a pathological driver, the cGAS-STING pathway has emerged as a promising therapeutic target, offering new avenues for intervening in various aging-related diseases and promoting healthy aging. Although current drug development efforts targeting this pathway have primarily focused on tumor immunity (agonists) and autoimmune diseases (inhibitors), with no agents yet approved for aging indications, its modulation has demonstrated considerable potential in preclinical aging models [28, 29]. This established role has galvanized the development of therapeutic strategies aimed at modulating the pathway. Current investigative approaches include specific cGAS/STING inhibitors (e.g., 30d-S, GHN105), interventions to mitigate DNA damage, and advanced nanoparticle-based delivery systems, all demonstrating considerable preclinical promise [30–33]. However, the translational application of these interventions still faces enduring challenges, particularly in terms of target specificity, long-term biosafety, and how to balance their physiological and pathological functions.

This review meticulously elucidates the role of the cGAS-STING pathway in cellular senescence, emphasizing its molecular mechanisms as they pertain to DNA damage response, paracrine senescence, hypoxic stress, ammonia-induced cell death, NET formation (NETosis), and macrophage polarization. We present a comprehensive analysis of the pathway's pathological contributions to a range of aging-related diseases affecting multiple systems, while critically assessing contemporary targeted intervention strategies and highlighting both advancements and ongoing challenges. By synthesizing state-of-the-art research findings, this review seeks to provide innovative insights into the immunometabolic regulatory network involved in senescence, and to lay a theoretical groundwork for the development of precision interventions aimed at combating aging and its associated pathologies.

## cGAS-STING, immunity, and senescence

The cGAS-STING signaling axis has emerged as a critical nexus integrating innate immune sensing with the establishment of cellular senescence [13]. This pathway is activated by aberrantly accumulated cytosolic DNA, including genomic DNA from damage or mitochondrial DNA (mtDNA) upon membrane permeabilization, triggering potent inflammatory signaling that drives senescence programs [17, 34]. Furthermore, cGAS-STING activation extends beyond cell-autonomous effects to orchestrate paracrine senescence by stimulating the SASP, thereby amplifying inflammatory responses both locally and systemically [35, 36]. This section will critically examine the multifaceted regulatory functions of the cGAS-STING pathway in processes such as the DNA damage response, paracrine senescence, hypoxic stress, ammonia-induced cell death, NETosis, and macrophage polarization, with the goal of delineating its complex mechanisms within the broader senescence network.

### The role of the cGAS-STING pathway in DNA damage and senescence

#### DNA damage-induced cGAS-STING activation triggers senescence

The genotoxic landscape of cellular senescence is shaped by a confluence of endogenous and exogenous insults. Endogenous sources include reactive oxygen species (ROS) [37], DNA replication errors [38], and topoisomerase-induced errors [39], while exogenous inducers encompass radiation [40], chemical agents [41], and viral infections [42]. Under physiological conditions, a robust network of DNA repair pathways—such as nucleotide excision repair (NER), base excision repair (BER), and non-homologous end joining (NHEJ)—efficiently resolves such damage to maintain genomic integrity [43–45]. A hallmark of senescence, however, is the significant downregulation of key DNA repair genes [45], creating a permissive environment for the accumulation of DNA lesions.

**Fig. 1:**
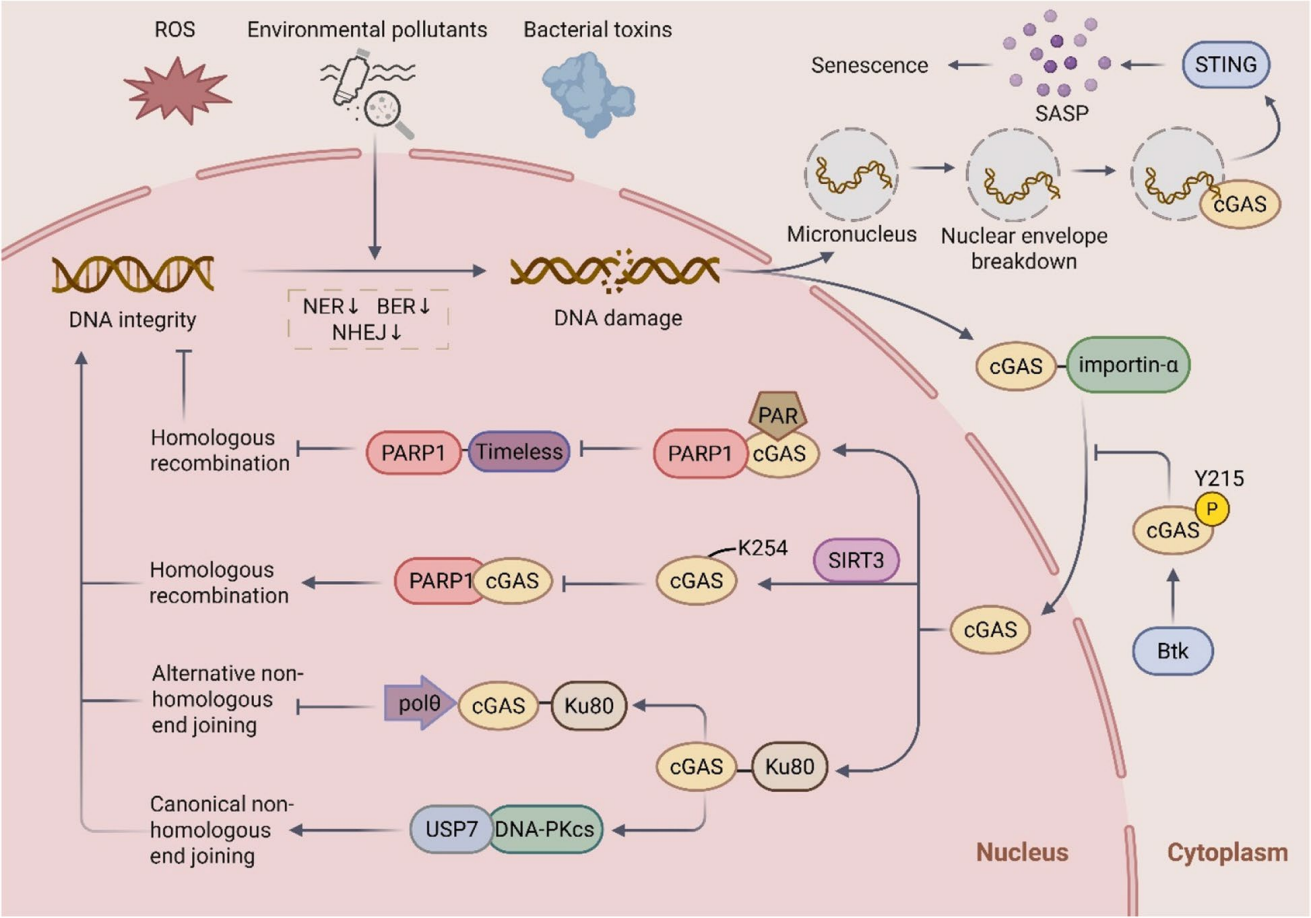
The mechanism by which cGAS regulates DNA damage response in cellular senescence. Senescence-related stimuli (such as reactive oxygen species [ROS], environmental pollutants, and bacterial toxins) induce DNA damage, leading to micronucleus formation and nuclear membrane rupture. This results in the release of double-stranded DNA (dsDNA) into the cytoplasm, where it binds to cGAS and activates the STING signaling pathway, promoting the production of senescence-associated secretory phenotype (SASP) and ultimately triggering cellular senescence. During this process, the functionality of DNA repair pathways—including nucleotide excision repair (NER), base excision repair (BER), and non-homologous end joining (NHEJ)—is downregulated, further exacerbating DNA damage. DNA damage induces the nuclear translocation of cytoplasmic cGAS in an importin-α-dependent manner, while Btk-mediated phosphorylation of cGAS at tyrosine 215 (Y215) promotes its cytoplasmic retention. In the nucleus, cGAS binds to PARP1 via poly (ADP-ribose) (PAR), inhibiting the formation of the PARP1-Timeless complex and thereby suppressing homologous recombination (HR) repair. Conversely, SIRT3-mediated decrotonylation of cGAS at lysine 254 (K254) disrupts this interaction, promoting HR repair. Additionally, nuclear cGAS forms a complex with Ku80, which (1) suppresses Polθ transcription to inhibit alternative NHEJ (alt-NHEJ) and (2) enhances the interaction between DNA-PKcs and the deubiquitinase USP7 to stabilize DNA-PKcs, thereby promoting classical NHEJ (c-NHEJ) and maintaining genomic integrity

This persistent genotoxic stress promotes the leakage of DNA into the cytosol, with micronuclei (MN) formation representing a primary mechanism [37, 46]. Genomic instability leads to chromosome missegregation and the encapsulation of lagging chromatin fragments within MN. A critical subsequent event is the frequent rupture of the micronuclear envelope, which exposes self-DNA to the cytosol [47, 48]. This exposed DNA acts as a potent ligand for the cytosolic sensor cGAS, leading to its robust recruitment and the activation of STING-dependent interferon signaling, thereby coupling chromosomal instability to innate immune activation [49] (Fig. 1). Intriguingly, not all MN are equally immunogenic. Studies indicate that MN generated by ionizing radiation or replication stress often contain cGAS that is bound to DNA but fails to initiate downstream signaling [50]. This suggests that chromatin structure within MN, potentially involving histones that sterically hinder cGAS activation, can impose a layer of regulatory control [50].

The convergence of DNA damage on cGAS-STING signaling establishes a fundamental pathway driving senescence. For instance, exposure to polymethyl methacrylate (PMMA) nanoplastics (NPs) induces oxidative stress and impairs the NHEJ pathway in gastric epithelial cells, resulting in DNA damage and micronucleation [37]. This, in turn, activates the cGAS-STING pathway, culminating in a senescent phenotype marked by elevated senescence-associated β-galactosidase (SA-β-gal) activity and G1 cell cycle arrest [37]. Similarly, polyvinyl chloride (PVC) NPs activate cGAS-STING and its downstream effector NF-κB, upregulating inflammatory mediators to promote senescence [34]. However, PVC NPs appear to operate through a distinct mechanism, suppressing homologous directed repair (HDR) by inhibiting the expression of breast cancer susceptibility gene 2 (BRCA2) and growth factor receptor-bound protein 2 (GRB2) [34]. This contrast highlights the stimulus-specific nature of DNA damage responses and the complexity of the ensuing senescence program. Beyond environmental pollutants, bacterial genotoxins also exploit this pathway. The *Salmonella typhi* typhoid toxin causes mtDNA damage, generating mitochondrial ROS (mtROS) that prompts the release of mtDNA into the cytosol [17]. The cytosolic mtDNA then engages the cGAS-STING axis to instigate senescence and the pro-inflammatory SASP in target cells [17]. Collectively, these findings solidify the role of the cGAS-STING pathway as a central mechanistic bridge, translating diverse genotoxic stresses into the execution of cellular senescence.

#### The regulatory impact of cGAS on the DNA damage response influences senescence

The canonical role of cGAS as a cytosolic DNA sensor that activates STING to initiate innate immune responses is well-established [51, 52]. However, emerging research has unveiled critical non-canonical functions for cGAS within the nucleus, expanding its role beyond immune signaling to direct regulation of genomic integrity. Following genotoxic stress, cGAS undergoes importin-α-dependent nuclear translocation [53]. Within the nucleus, cGAS interacts with PARP1 via poly(ADP-ribose) chains, thereby antagonizing homologous recombination (HR) repair by disrupting the PARP1-Timeless complex [53, 54]. The subcellular localization of cGAS is a key regulatory point: phosphorylation at tyrosine 215 by B-lymphoid tyrosine kinase promotes its retention in the cytosol [53]. Furthermore, SIRT3-mediated decrotonylation of cGAS at lysine 254 reduces its DNA-binding affinity and interaction with PARP1, effectively promoting HR repair [55]. This regulatory interplay suggests that nuclear cGAS exerts a dual role in fine-tuning DNA repair pathways.

In addition to HR, nuclear cGAS can also regulate other DNA double-strand break (DSB) repair pathways. The cGAS-Ku80 complex constitutes a key regulatory module that differentially governs canonical (c-NHEJ) and alternative (alt-NHEJ) subpathways [56–59]. It promotes the more accurate c-NHEJ by enhancing the DNA-PKcs-USP7 interaction to stabilize DNA-PKcs, while concurrently suppressing the error-prone alt-NHEJ through direct binding to the Polθ promoter to repress its transcription [59] (Fig. 1). Additionally, nuclear cGAS maintains genomic stability by suppressing LINE-1 (L1) retrotransposition, a function that is critical for the senescence-associated suppression of L1 activity [60]. Collectively, these findings position nuclear cGAS as a multifaceted guardian of the genome that not only safeguards genomic integrity but also exerts precise control over the DNA damage response (DDR), thereby influencing DNA damage-induced cellular senescence. It is important to note that the persistence and functional activity of cGAS in the nucleus may be context-dependent, varying with cell type and specific stress conditions, an area that warrants further clarification.

The relationship between the cGAS-STING pathway and senescence extends to telomeres, the protective caps of eukaryotic chromosomes that are central to the DDR and cellular senescence [61, 62]. Telomere shortening or dysfunction can lead to chromosomal instability, generating cytoplasmic telomeric fragments that activate the cytosolic cGAS-STING pathway and drive a pro-senescent inflammatory response [63, 64]. Intriguingly, nuclear cGAS also directly modulates telomere homeostasis. Telomeres are protected by the shelterin complex, but during mitosis, cGAS can occupy shelterin-deficient telomeric and subtelomeric regions, binding to exposed telomeric DNA [65]. In this capacity, cGAS recruits CDK1 to chromosomal ends, an interaction that inhibits telomeric DDR and prevents c-NHEJ-mediated telomere fusion. The absence of this protective mechanism leads to end-to-end fusions, which paradoxically dampen DDR at critically short telomeres and enable cells to bypass replicative senescence [65]. Thus, nuclear cGAS-mediated inhibition of telomere fusion is essential for the activation of replicative senescence. While the cytoplasmic role of cGAS-STING in responding to dysfunctional telomeres is clear, the direct regulatory functions of nuclear cGAS at telomeres and their precise contribution to senescence pathways present a compelling avenue for future research.

### The release of inflammatory factors mediated by cGAS-STING signaling promotes paracrine senescence

The accumulation of cytoplasmic DNA in senescent cells serves as a potent trigger for the cGAS-STING signaling pathway. This activation initiates a robust inflammatory response characterized by the secretion of numerous soluble factors, which not only reinforces the cell-autonomous senescent state but also propagates senescence to neighboring cells through paracrine mechanisms [10, 16, 66]. This phenomenon, termed paracrine senescence, expands the traditional concept of cellular senescence beyond cell-autonomous effects to encompass its systemic impact on tissue homeostasis and organismal aging [67–69]. Paracrine senescence is primarily orchestrated by SASP, a complex repertoire of bioactive molecules—including cytokines, chemokines, and proteases—released by senescent cells [35, 70, 71]. Given its role in driving aging and aging-related pathologies, targeting the paracrine actions of senescent cells has emerged as a major therapeutic strategy.

The cGAS-STING pathway is a principal regulator of SASP induction across diverse cell types [72–74]. For instance, in typhoid toxin-induced senescence, cytosolic mtDNA activates cGAS-STING signaling in macrophages, leading to SASP release [17]. Exposure to this SASP-rich conditioned medium is sufficient to induce paracrine senescence in CD4^+^ T cells, as evidenced by G2/M cell cycle arrest and elevated SA-β-gal activity [17]. This transmissible nature of senescence is further demonstrated in vivo by the observation that transplanted senescent cells can induce senescence in surrounding host tissues [75]. However, the SASP is subject to contextual regulation; for example, monoacylglycerol lipase (MGLL) overexpression in senescent prostate tumor cells suppresses NF-κB-driven SASP, thereby attenuating both autocrine and paracrine tumor-promoting effects [76]. These findings underscore that targeting cGAS-STING-driven SASP may offer a viable approach to curbing the propagation of senescence.

**Fig. 2:**
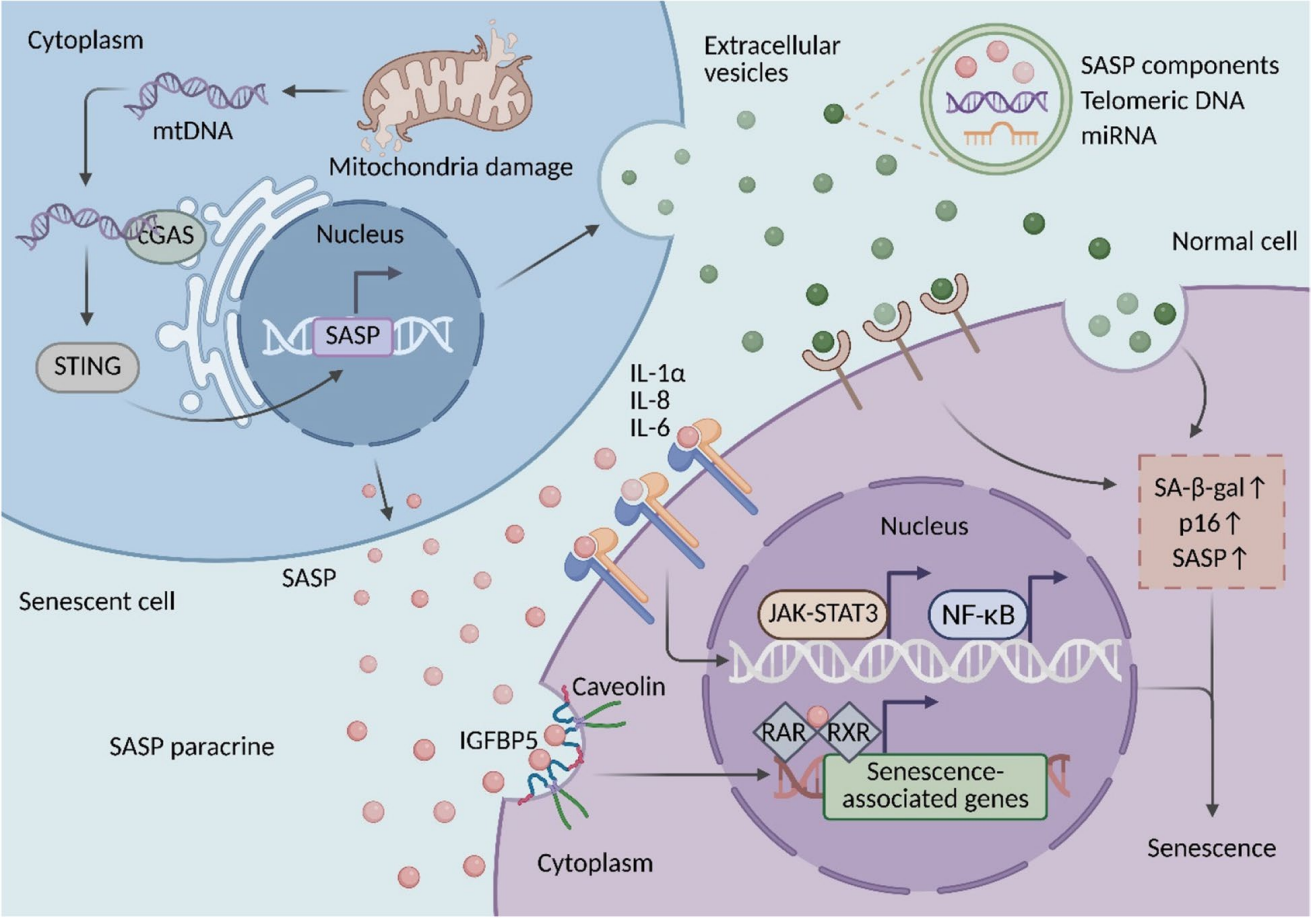
Mechanisms of paracrine senescence mediated by SASP in senescent cells. In senescent cells, mitochondrial damage leads to mtDNA leakage into the cytosol, activating the cGAS-STING pathway and subsequently upregulating the expression of senescence-associated secretory phenotype (SASP) genes in the nucleus. Secreted SASP factors (e.g., IL-1α, IL-6, and IL-8) bind to membrane receptors on neighboring cells, activating signaling pathways such as JAK-STAT3 and NF-κB. Meanwhile, other SASP components like IGFBP5 enter the nucleus via caveolin-dependent endocytosis, where they interact with the RAR/RXR heterodimer to regulate the transcription of senescence-related genes. Additionally, extracellular vesicles (EVs) serve as critical delivery vehicles, carrying SASP components, telomeric DNA fragments, and miRNAs. These EVs are internalized by recipient cells through receptor binding or endocytosis, promoting increased SA-β-gal activity, elevated p16 expression, and accumulation of SASP markers. Together, these mechanisms synergistically amplify senescence signals through paracrine pathways, ultimately driving the propagation of the senescent phenotype in neighboring cells

A critical mechanism for SASP-mediated intercellular communication involves extracellular vesicles (EVs), which act as carriers for a subset of SASP components [77, 78]. Senescent cholangiocytes, for example, release increased levels of EVs that propagate paracrine senescence in normal human cholangiocytes, characterized by elevated SA-β-gal activity, p16 expression, and SASP marker levels [18]. Similarly, EVs from physiologically senescent bone marrow monocytes/macrophages can mediate systemic senescence in mice [79]. Beyond soluble factors, EVs can transport genomic materials; senescent cells secrete EVs carrying telomeric DNA fragments, which are capable of inducing senescence in recipient cells, as demonstrated in Tcea1-deficient mouse embryonic fibroblasts [80]. Exosomes, a specific subclass of EVs, are particularly enriched in microRNAs (miRNAs) and serve as efficient vehicles for senescence amplification [81]. For instance, infection of murine dendritic cells with *Porphyromonas gingivalis* triggers a SASP comprising both cytokines and exosomes [82]. These exosomes efficiently transmit the senescent state to bystander dendritic and T cells, highlighting their role as potent transmitters of paracrine senescence (Fig. 2).

In addition to EV-mediated communication, soluble SASP factors can directly activate membrane receptors on neighboring cells to drive paracrine senescence. In mesenchymal stromal cells (MSCs), SASP components such as IL-1α and IL-8 from late-passage, senescent MSCs induce a senescent phenotype in early-passage MSCs via activation of the NF-κB pathway; inhibition of NF-κB signaling ablates this effect [35]. Similarly, in nucleus pulposus cells, TNF-α-induced senescent cells drive the paracrine senescence of healthy cells through a mechanism involving phosphorylated STAT3 (p-STAT3) and the key cytokine IL-6 [83]. A feed-forward loop is observed in glioblastoma (GBM), where senescent glioma cells secrete IL-6, which concurrently activates the JAK-STAT3 and NF-κB pathways in adjacent non-senescent cells, thereby amplifying both tumor proliferation and the SASP [36]. Recently, a non-canonical mechanism has been identified for insulin-like growth factor binding protein 5 (IGFBP5), a SASP component that induces paracrine senescence in cells not directly exposed to genotoxic stress [84]. Mechanistically, IGFBP5 enters the nucleus via caveolae-dependent endocytosis and interacts with the retinoic acid receptor/retinoid X receptor (RAR/RXR) heterodimer to modulate the transcription of senescence-associated genes [84] (Fig. 2).

In summary, the cGAS-STING pathway orchestrates SASP production, which in turn drives paracrine senescence through mechanisms involving EVs and receptor signaling. Notably, the repertoire of SASP factors mediating paracrine senescence exhibits significant heterogeneity across different physiological and pathological contexts. For instance, pro-inflammatory cytokines represented by IL-6 are consistently upregulated in various senescence models, potentially constituting a broad molecular foundation for paracrine senescence [36, 83, 85]. In contrast, factors such as IGFBP5 primarily function in specific contexts like stromal cell senescence, demonstrating clear cell-type and pathological context dependence [84, 86]. Therefore, future research urgently requires systematic cross-comparisons across diverse cell types and disease models to precisely delineate the landscape of key SASP factors driving paracrine senescence. Additionally, it is crucial to elucidate how the tissue microenvironment (e.g., hypoxia, immune infiltration) dynamically regulates SASP composition and functional output, for example, the central role of the IL-6/JAK-STAT3 axis in chronic inflammation. Systematic elucidation of these questions will constitute an essential foundation for developing precise, context-adaptive strategies for aging intervention.

### The cGAS-STING pathway is a master regulator of hypoxia-induced senescence

Hypoxia, a pathophysiological state defined by an inadequate oxygen supply, disrupts cellular metabolism and function. The cellular response to hypoxia is masterfully orchestrated by hypoxia-inducible factors (HIFs), with HIF-1α serving as the canonical marker of the hypoxic response [87–89]. Under normoxic conditions, HIF-1α is constitutively synthesized and rapidly degraded via the proteasome. In contrast, hypoxia stabilizes HIF-1α, allowing its nuclear translocation, heterodimerization with HIF-1β, and subsequent activation of a transcriptional program essential for adaptation [90]. A growing body of evidence positions hypoxia as a potent inducer of cellular senescence [91, 92]. Within the complex interplay between hypoxia and senescence, the cGAS-STING pathway has emerged as a pivotal innate immune sensing mechanism, integrating hypoxic stress into a senescent phenotype [93].

**Fig. 3:**
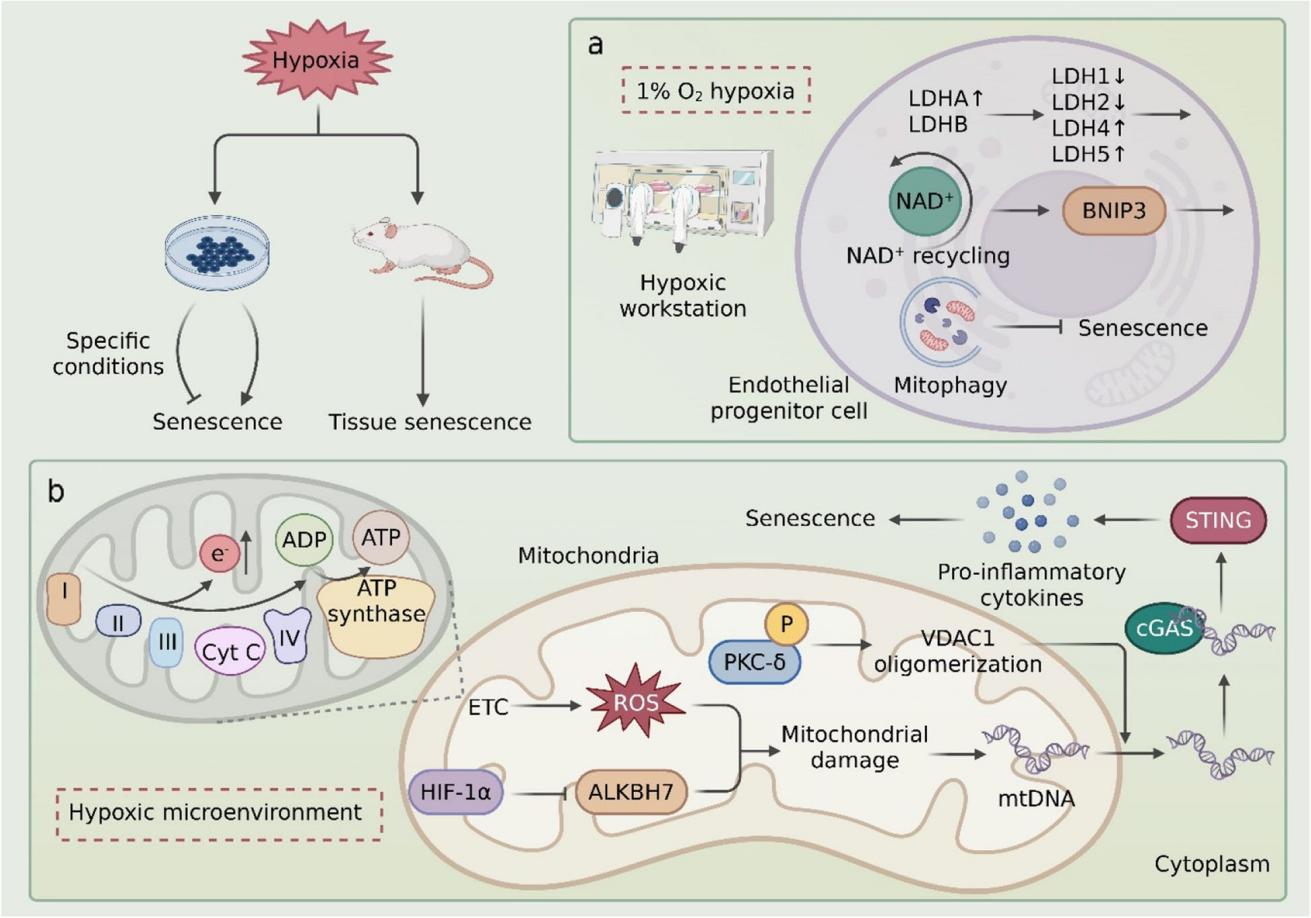
Hypoxia bidirectionally regulates cellular senescence through metabolic reprogramming and mitochondrial dysfunction. Hypoxia exerts dual regulatory effects on cellular senescence, with the outcome determined by microenvironmental conditions and cell type. **A:** Panel a illustrates that under 1% O₂ conditions, hypoxia induces lactate dehydrogenase (LDH) isoenzyme reprogramming (LDH1/2↓, LDH4/5↑) to enhance NAD⁺ cycling, while upregulating Bcl-2-interacting protein 3 (BNIP3)-mediated mitophagy, thereby maintaining redox homeostasis and delaying endothelial progenitor cell senescence. **B:** Panel b delineates the molecular mechanisms underlying hypoxia-induced senescence: (1) Increased electron leakage from the mitochondrial electron transport chain (ETC) leads to ROS accumulation; (2) HIF-1α-dependent downregulation of AlkB homolog 7 (ALKBH7) compromises mitochondrial integrity; (3) Phosphorylated PKC-δ facilitates voltage-dependent anion channel 1 (VDAC1) oligomerization to promote mtDNA release; (4) Cytosolic mtDNA activates the cGAS-STING pathway, inducing proinflammatory cytokine (IL-6/IFN-α) secretion and ultimately triggering a senescent phenotype

In vivo studies substantiate the role of hypoxia in promoting senescence. In wild-type mice, hypoxia elevates the pulmonary expression of senescence markers (p16, p21) and the DNA damage marker γ-H2AX [91]. Immunohistochemical analysis reveals increased p16 staining in pulmonary endothelial and smooth muscle cells, confirming senescence in these specific populations [91]. Similarly, chronic intermittent hypoxia (CIH) induces a premature senescence phenotype in the epididymal white adipose tissue (eWAT) of mice, characterized by a heightened DNA damage response, inflammation, and upregulation of p16, p21, and p53 [92]. The involvement of HIF-1α in this process is often critical. For instance, the mycotoxin T-2 induces senescence in human neuroblastoma cells concomitant with HIF-1α upregulation, where HIF-1α potentiates the induction of p16, p21, and p53, exacerbating cell cycle arrest [94]. Furthermore, HTRA1, a major genetic risk factor for age-related macular degeneration (AMD), induces cellular hypoxia and activates HIF-1 signaling in retinal pigment epithelial cells, thereby driving senescence—an effect that can be rescued by the HIF-1α inhibitor KC7F2 [95]. However, the relationship between hypoxia and senescence is not universally linear; under specific conditions, hypoxia can paradoxically attenuate senescence. For example, in endothelial progenitor cells (EPCs), 1% O₂ hypoxia induces a metabolic shift in lactate dehydrogenase (LDH) isozymes, promoting nicotinamide adenine dinucleotide (NAD⁺) recycling, upregulating the mitophagy receptor BNIP3, and ultimately reducing senescence [96] (Fig. 3). This apparent paradox underscores the context-dependency of hypoxic signaling, influenced by factors such as cell type, duration (acute vs. chronic), and microenvironment. Delineating the mechanisms underlying these divergent outcomes may yield novel therapeutic avenues for aging-related diseases and cancer.

Given the established role of DNA damage and the cGAS-STING pathway in senescence, investigating its involvement in hypoxia-mediated senescence has become a compelling research direction. Multiple lines of evidence confirm that hypoxia activates the cGAS-STING pathway [97, 98]. In macrophages, the T-2 toxin induces immunosenescence by activating the HIF-1α/cGAS-STING axis, leading to the upregulation of pro-senescent factors like IL-6, IL-8, and CCL-2 [93]. Intriguingly, HIF-1α may exert an initial negative regulatory effect in this model, highlighting the dynamic nature of its involvement [93]. Mitochondria, as highly oxygen-sensitive organelles, are central to this connection. Hypoxia perturbs the mitochondrial electron transport chain (ETC), increasing electron leakage and generating excessive ROS that damage mtDNA and facilitate its release into the cytosol [99, 100]. This mechanism is operative in various cell types. In adenomyosis (AM) endometrial stromal cells (ESCs), hypoxia-induced cytosolic mtDNA leakage activates cGAS-STING, driving the production of IL-6 and IFN-α [99]. Similarly, in fibroblast-like synoviocytes, hypoxia triggers mitochondrial dysfunction and cGAS-STING activation, promoting a pro-inflammatory phenotype [101]. This process is mediated by HIF-1α through the targeted downregulation of the mitochondrial protein ALKBH7 [101]. Additional regulatory nodes are also involved. Protein kinase C-delta (PKC-δ) acts as a key mediator in HK-2 cells, where hypoxia triggers its phosphorylation [19]. Activated PKC-δ then promotes the oligomerization of the voltage-dependent anion channel 1 (VDAC1) on the mitochondrial membrane, facilitating mtDNA release and subsequent cGAS-STING pathway activation [19] (Fig. 3).

In summary, a sophisticated regulatory network connects hypoxic stress to cellular senescence, with the cGAS-STING pathway serving as a crucial signaling hub. Hypoxia, often via HIF-1α, induces mitochondrial dysfunction and mtDNA release, which in turn activates cGAS-STING to promulgate a senescent and inflammatory phenotype. Despite these advances, the field is still nascent. Key questions remain, including the precise mechanistic contributions of cGAS-STING in different hypoxic contexts, the determinants of its pro- versus anti-senescence outcomes, and the potential for therapeutic targeting of this axis to mitigate aging and disease.

### The possible involvement of cGAS-STING in ammonia-induced cell death and senescence

Ammonia-induced cell death is a recently identified form of programmed cell death triggered by the excessive accumulation of ammonia (NH₃/NH₄⁺) [22]. As a metabolic waste product generated from processes such as amino acid catabolism and glutaminolysis, ammonia disrupts cellular homeostasis at elevated concentrations, inducing pH imbalance, mitochondrial dysfunction, oxidative stress, and endoplasmic reticulum stress [102–105]. While ammonia toxicity, cGAS-STING signaling, and cellular senescence have been intensively studied within their respective domains of metabolic disorders, innate immunity, and aging, emerging evidence suggests a potential intersection along a metabolic-immuno-senescence axis. Elucidating the crosstalk between these pathways may reveal novel mechanistic links connecting metabolic stress to immune activation and senescence, offering fresh perspectives on the pathogenesis of degenerative conditions such as hepatic encephalopathy and Alzheimer's disease.

**Fig. 4:**
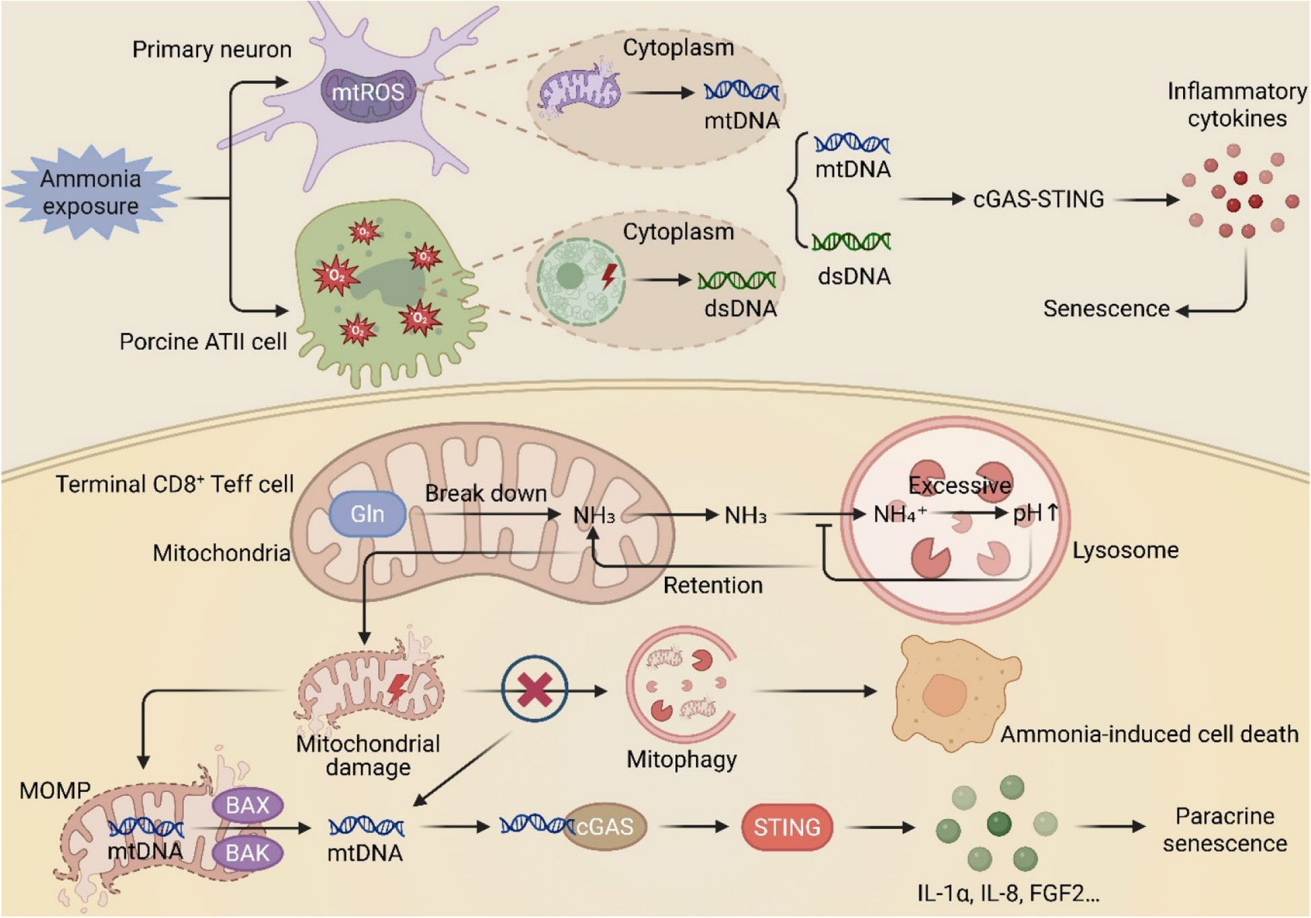
Mitochondrial ammonia metabolic disorder triggers mitochondrial damage and cellular senescence via the cGAS-STING pathway. In terminal CD8 + effector T cells (Teff), ammonia generated from mitochondrial glutamine (Gln) catabolism is transported to lysosomes for storage. However, excessive accumulation elevates lysosomal pH, leading to ammonia retention within mitochondria and causing severe damage. Such damaged mitochondria cannot be cleared via mitophagy, ultimately resulting in ammonia-induced cell death. We speculate that these impaired mitochondria undergo mitochondrial outer membrane permeabilization (MOMP) due to BAX/BAK oligomerization, forming large pores that hinder mitophagy clearance while promoting mtDNA release into the cytoplasm. The accumulated mtDNA persistently activates the cGAS-STING pathway, inducing the secretion of SASP factors such as IL-1α, IL-8, and FGF2, thereby promoting paracrine senescence. Similar mechanisms are observed in other cell types: in neurons, ammonia exposure triggers mitochondrial ROS bursts, leading to mitochondrial dysfunction; in alveolar type II epithelial cells (ATII), ammonia induces oxidative stress, causing chromatin condensation, nuclear fragmentation, and DNA oxidative damage. These processes may lead to the leakage of mtDNA from damaged mitochondria and dsDNA from the nucleus, further activating the cGAS-STING pathway to trigger inflammatory responses and accelerate cellular senescence

The characterization of ammonia-induced cell death in rapidly proliferating effector CD8^+^ T cells provides a compelling model for its interplay with mitochondrial integrity and immune signaling [22]. In these cells, mitochondrial glutaminolysis supports proliferation but concurrently releases ammonia, which is initially sequestered in lysosomes. Upon exceeding lysosomal storage capacity, ammonia accumulates within mitochondria, leading to severe ultrastructural damage, including disruption of the inner membrane and cristae [22]. Critically, this damage coincides with a failure of mitophagy, preventing the clearance of compromised organelles and ultimately triggering cell death. This failure to clear damaged mitochondria is a known driver of cellular senescence [106, 107]. Mitochondrial membrane damage promotes BAX/BAK oligomerization and mitochondrial outer membrane permeabilization (MOMP), facilitating the release of mtDNA into the cytosol [108, 109]. When coupled with impaired mitophagy, this results in the persistent accumulation of cytosolic mtDNA [110, 111]. This misplaced mtDNA serves as a potent ligand for the cytosolic DNA sensor cGAS, activating the STING pathway and downstream production of IFN-Is and SASP factors [52, 112, 113]. Notably, key SASP components induced by cGAS-STING activation, such as IL-1α, IL-8, and FGF2, are established mediators of paracrine senescence [35, 114]. Therefore, a plausible model emerges wherein ammonia-induced mitochondrial damage triggers mtDNA-dependent cGAS-STING activation, leading to the secretion of SASP factors that can propagate senescence to neighboring cells (Fig. 4).

Beyond the context of T cell biology, ammonia toxicity is a hallmark of hyperammonemic conditions such as hepatic failure [115, 116]. In rats with bile duct ligation (BDL)-induced cirrhosis, elevated plasma ammonia leads to mtROS overproduction in the brain [117]. Similarly, NH₄Cl treatment induces mtROS and oxidative stress in primary neurons [117]. Given that mtROS is a well-established cause of mitochondrial damage and subsequent mtDNA release—as demonstrated in copper-exposed astrocytes where it activates cGAS-STING [118]—it is reasonable to speculate that ammonia-induced mtROS contributes to cytosolic mtDNA leakage and innate immune activation. In addition to mtDNA, nuclear-derived dsDNA in the cytoplasm is a potent activator of the cGAS-STING pathway [37, 119]. In vitro models using NH₄Cl to simulate hyperammonemia demonstrate that ammonia exposure induces significant oxidative stress [116]. In porcine intestinal epithelial (IPEC-J2) cells, NH₄Cl treatment increases ammonia uptake and intracellular ROS, while reducing mitochondrial membrane potential [120]. Elevated ROS can induce nuclear DNA instability, leading to strand breaks [121, 122]. Supporting this, ammonia exposure in porcine alveolar type II epithelial cells (ATII) causes oxidative stress, chromatin condensation, nuclear fragmentation, and DNA oxidative damage, while simultaneously suppressing the expression of DNA repair genes [23]. While low-level ROS may cause reparable damage, sustained high-intensity oxidative stress can overwhelm repair mechanisms, potentially leading to the leakage of nuclear DNA into the cytosol via pathways such as nuclear membrane disruption or micronucleus formation. Notably, emerging evidence suggests that hyperammonemia leads to dysregulation of cellular redox status, driving cellular senescence by inhibiting sirtuin-mediated deacetylation [123]. This senescent phenotype closely parallels the downstream effects of chronic cGAS-STING pathway activation, phenotypically supporting a potential link between ammonia exposure, aberrant innate immune activation, and the aging process **(**Fig. 4**)**.

Although direct experimental evidence causally linking ammonia-induced cell death to cGAS-STING pathway activation and senescence remains insufficient, the association is supported by a rational mechanistic foundation and indirect evidence. Ammonia exposure triggers mitochondrial damage, ROS burst, and oxidative DNA damage, which are established upstream events known to cause cytosolic DNA leakage and activate the cGAS-STING pathway [22, 117]. In addition, the senescent-like phenotypes and upregulation of inflammatory factors observed in ammonia toxicity models closely parallel the consequences of cGAS-STING overactivation [123]. However, definitive validation of this axis requires key experimental evidence. This includes direct detection of cytosolic DNA (particularly mtDNA) accumulation, elevated cGAMP levels, and STING phosphorylation following ammonia exposure. Genetic or pharmacological interventions are also needed to establish the necessity of this pathway in mediating the associated senescent phenotypes. Future studies are warranted to fill these evidence gaps in cellular and hyperammonemia disease models, thereby elucidating the pathophysiological significance of this metabolic-immune-senescence axis.

### The NET-cGAS-STING-senescence axis: a self-amplifying pathological circuit

Extracellular Traps (ETs) are web-like structures composed of decondensed chromatin, histones, and antimicrobial proteins, which are released by activated immune cells, including neutrophils, macrophages, and eosinophils, as a defense mechanism to ensnare pathogens [124–127]. While ET formation is a crucial component of innate immunity, its dysregulation and persistent release are increasingly implicated in the pathology of chronic inflammatory and aging-related diseases [124, 128]. Among the various ET subtypes, NETs are the most extensively characterized. A growing body of evidence reveals a dynamic interplay between NETs, the cGAS-STING pathway, and senescent cells, forming a pathogenic axis that fuels sterile inflammation and tissue degeneration.

**Fig. 5:**
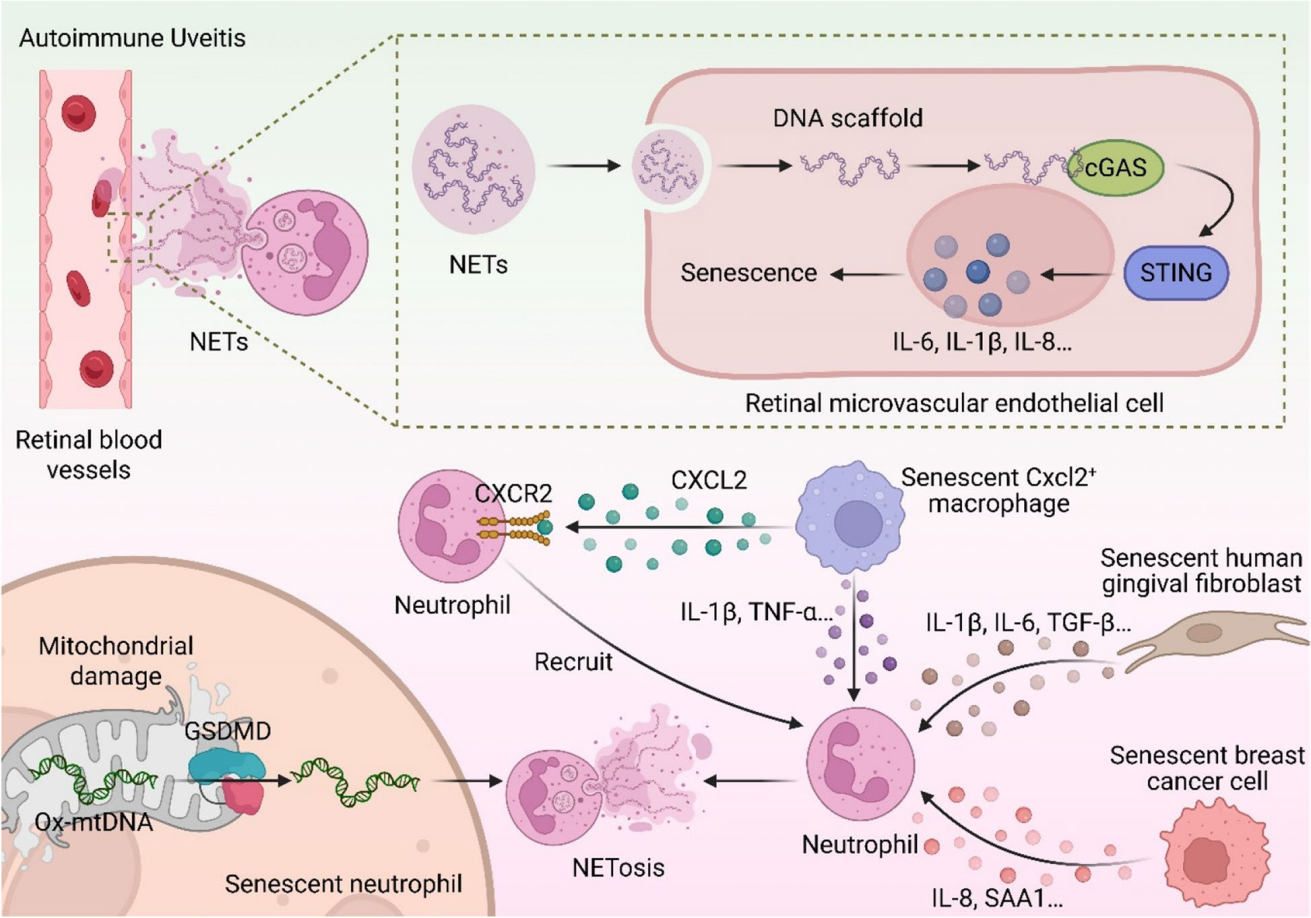
Positive feedback regulatory mechanism between the NETs-cGAS-STING pathway and cellular senescence. In autoimmune uveitis (AU), neutrophil aggregation around retinal blood vessels is enhanced, accompanied by NETs formation. NETs consist of a DNA scaffold embedded with bactericidal and vasoactive proteins, which can be taken up by retinal microvascular endothelial cells (RMECs) via endocytosis. The internalized NETs escape into the cytoplasm and activate the cGAS-STING pathway through their DNA scaffold, inducing the production of cytokines such as IL-6, IL-1β, and IL-8, thereby promoting cellular senescence. Notably, SASP factors generated by cGAS-STING pathway activation can feedback to promote NETosis: senescent Cxcl2 + macrophages recruit neutrophils via the CXCL2-CXCR2 axis and secrete pro-NETosis factors; senescent human gingival fibroblasts significantly enhance NETosis through SASP factors (e.g., IL-1β, IL-6, TGF-β); and senescent breast cancer cells recruit neutrophils and activate NETosis via IL-8 and SAA1. Additionally, senescent neutrophils themselves exhibit increased susceptibility to NETosis, which may be related to senescence-associated mitochondrial damage—mitochondrial damage leads to GSDMD-mediated translocation of oxidized mtDNA (Ox-mtDNA) to the cytoplasm, thereby triggering Ox-mtDNA-containing NETosis

In numerous pathological contexts, massive immune cell infiltration leads to abundant ET release [129, 130]. NETs, in particular, have been demonstrated to activate the cGAS-STING pathway, thereby instigating inflammatory cytokine production and cellular senescence. In autoimmune uveitis (AU), for instance, pronounced neutrophil accumulation and NETosis are observed in perivascular regions of the retina [20, 131]. Retinal tissues from experimental AU (EAU) mice exhibit an increased prevalence of SA-β-gal-positive cells colocalized with elevated inflammatory cytokines (e.g., IL-6, IL-1β, IL-8), suggesting NETs promote senescence in retinal microvascular endothelial cells (RMECs) [20]. Mechanistic studies confirm that NETs directly trigger cGAS-STING activation in RMECs, establishing a causal link to the senescent phenotype. The pathological significance of this cascade is further underscored in acute lung injury (ALI) models, where Icariside II (ICS II), a natural compound with anti-inflammatory properties, ameliorates disease by inhibiting NETosis via targeting of neutrophil CXCR4 [132–134]. This reduction in NETs subsequently suppresses cGAS/STING/NF-κB pathway activation and inflammatory mediator production in pulmonary epithelial cells. The activation of cGAS-STING by NETs is primarily mediated by their DNA scaffold [125, 135]. Internalization of NETs by target cells, such as macrophages and alveolar epithelial cells (AECs), occurs via endocytosis. A fraction of this internalized NET-derived DNA subsequently escapes into the cytosol, where it serves as a potent ligand for cGAS, driving the production of IFN-Is and other proinflammatory factors like TNF-α [136, 137] (Fig. 5). Despite these advances, key mechanistic questions persist, including the precise molecular basis by which cGAS distinguishes NET-associated self-DNA from foreign DNA, and whether alternative routes for cytosolic DNA delivery exist beyond endocytosis.

The relationship between NETs and senescence is reciprocal. While NETs can induce senescence, SASP generated by senescent cells—whether through NET-induced cGAS-STING activation or other triggers—can, in turn, potentiate NETosis, creating a feed-forward loop of inflammation [138, 139]. For example, senescent human gingival fibroblasts secrete a SASP rich in IL-1β, IL-6, and TGF-β, and their conditioned medium robustly enhances NETosis [140]. In aged livers, a subset of Cxcl2^+^ macrophages with a SASP recruits neutrophils via the CXCL2-CXCR2 axis and promotes NETosis through IL-1β and TNF-α secretion [141]. Similarly, therapy-induced senescent breast cancer cells develop an inflammatory secretome that recruits and activates neutrophils, partly via IL-8 and acute-phase serum amyloid A1 (SAA1), leading to NET release [139]. This propensity for NETosis is further amplified in senescent neutrophils themselves. Senescent neutrophils exhibit a heightened susceptibility to NETosis upon stimulation with agents like lipopolysaccharide (LPS) [142]. The underlying mechanism may involve senescence-associated mitochondrial dysfunction. In neutrophils from high-fat diet (HFD) models, mitochondrial damage leads to gasdermin D (GSDMD)-pore-mediated cytosolic leakage of oxidized mtDNA (ox-mtDNA), which activates the cGAS-STING pathway and drives the release of ox-mtDNA-containing NETs [143, 144]. Given that mitochondrial impairment is a hallmark of senescence, it is plausible that this cGAS-STING-dependent mechanism contributes to the hyper-NETotic state of senescent neutrophils (Fig. 5). Crucially, pharmacological inhibition of cGAS-STING signaling suppresses this mitochondrial damage-induced NETosis, confirming the pathway's pivotal role [143].

In summary, NETs, the cGAS-STING pathway, and cellular senescence are enmeshed in a self-perpetuating, pathogenic cycle. NET-derived DNA activates cGAS-STING signaling, driving both local inflammation and paracrine senescence. The ensuing SASP from affected cells then recruits and primes neutrophils, further amplifying NETosis. This vicious cycle highlights a critical nexus between innate immunity and the biology of senescence. Future research must focus on delineating the specific SASP factors that are key drivers of NETosis and evaluating therapeutic strategies that disrupt this axis, offering promising avenues for treating a spectrum of chronic inflammatory and aging-related diseases.

### cGAS-STING and senescence: the key players in macrophage polarization

Macrophages, as central sentinels of the innate immune system, exhibit remarkable functional plasticity, dynamically altering their phenotype in response to microenvironmental signals—a process known as polarization [140, 145, 146]. This adaptability gives rise to a spectrum of activation states, most commonly categorized into classically activated (M1) pro-inflammatory macrophages and alternatively activated (M2) anti-inflammatory or reparative macrophages [147, 148]. The precise balance between M1 and M2 polarization is a critical determinant of outcomes in inflammation, infection, cancer, and metabolic diseases [149, 150]. Emerging evidence now positions the cGAS-STING signaling pathway and cellular senescence as significant regulators of this equilibrium [21, 151, 152]. Elucidating the mechanistic interplay between these elements holds considerable promise for developing novel therapeutic strategies for cancer, aging-related pathologies, and fibrotic disorders.

**Fig. 6:**
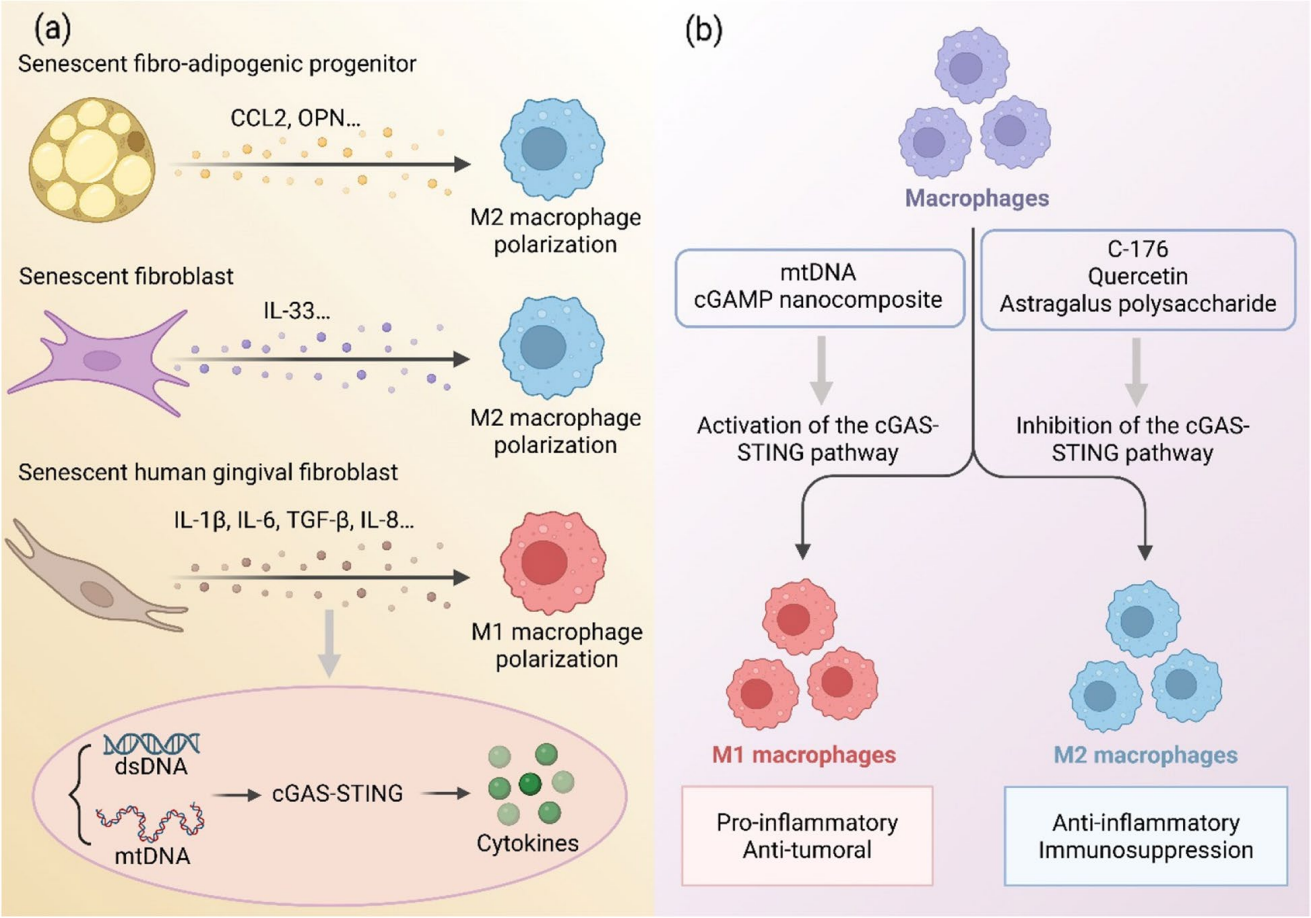
Senescent cells regulate macrophage polarization through secretome and cGAS-STING pathway. **A** Regulation of macrophage polarization by senescent cell secretome. Senescent cells promote macrophage recruitment and polarization through the secretion of various cytokines. For example, senescent fibro-adipogenic progenitors (FAPs) secrete CCL2 and osteopontin (OPN, encoded by Spp1), which significantly enhance macrophage recruitment and drive their polarization toward the M2 phenotype. Senescent fibroblasts produce the key SASP factor IL-33, which strongly promotes M2 polarization. Senescent human gingival fibroblasts (I-GFs) exhibit significantly elevated expression of SASP factors (IL-1β, IL-6, TGF-β, and IL-8), and their conditioned medium induces macrophage polarization toward the pro-inflammatory M1 phenotype. Additionally, the accumulation of cytoplasmic DNA in senescent cells activates the cGAS-STING pathway, leading to cytokine secretion, suggesting that this pathway may partially mediate senescent cell regulation of macrophage polarization. **B** Direct regulation of polarization by intrinsic cGAS-STING signaling in macrophages. Beyond the paracrine effects of senescent cells, the activity of the cGAS-STING pathway in macrophages directly influences the M1/M2 polarization balance: Activation of cGAS-STING (e.g., by mtDNA or cGAMP nanocomplexes) promotes M1 polarization, resulting in a pro-inflammatory phenotype and anti-tumor effects, whereas inhibition of cGAS-STING (e.g., by C176, quercetin, or astragalus polysaccharides) promotes M2 polarization, leading to an anti-inflammatory phenotype and immunosuppressive functions

Senescent cells exert a profound influence on the immune landscape through their SASP, which includes a repertoire of cytokines and chemokines that recruit and polarize macrophages [77, 153]. The specific composition of the SASP dictates the resulting macrophage phenotype. For instance, in aged murine skeletal muscle, senescent fibro-adipogenic progenitors (FAPs) secrete CCL2 and osteopontin (OPN), which drive macrophage recruitment and polarization toward an M2-like, pro-reparative state [151]. Similarly, IL-33, a key SASP factor from senescent fibroblasts, promotes M2 polarization to facilitate tissue repair in models of radiation-induced skin injury [154]. Conversely, senescent human gingival fibroblasts from inflamed tissues (I-GFs), which produce a SASP rich in IL-1β, IL-6, TGF-β, and IL-8, promote polarization toward a CD86-positive M1 pro-inflammatory phenotype [140]. Parallel to senescence, the cGAS-STING pathway is a potent driver of macrophage polarization [155, 156]. In multiple myeloma, cGAS-STING activation in tumor cells induces M1 polarization of surrounding macrophages [152]. Furthermore, in radiation-induced lung injury (RILI), the cGAS-STING pathway is essential for pulmonary macrophage recruitment and polarization; its deficiency impairs this response, an effect reversible by CCL2 antagonism, highlighting a key mechanistic role for this chemokine [157]. A notable paracrine mechanism involves irradiated cancer cells, where cytosolic mtDNA accumulation activates cGAS-STING, leading to IFN-β secretion that induces M1 polarization in distant macrophages via STAT1 signaling [158]. Given that senescent cells also accumulate cytoplasmic DNA and activate cGAS-STING to generate a SASP, it is plausible that senescent cells regulate macrophage polarization, at least in part, through this pathway (Fig. 6). However, potential crosstalk with other innate immune pathways, such as TLRs or the NLRP3 inflammasome, warrants further investigation.

Beyond paracrine effects, the intrinsic activity of the cGAS-STING pathway within macrophages directly governs their polarization fate. Generally, pathway activation skews macrophages toward a pro-inflammatory M1 phenotype, while its inhibition favors the M2 anti-inflammatory state [159–161]. For example, in diabetic wounds, high glucose-induced ROS promote mtDNA-dependent cGAS-STING activation, driving deleterious M1 polarization [21]. Therapeutic interventions corroborate this paradigm: delivering the STING inhibitor C176 via nanoparticles to retinal macrophages suppresses cGAS-STING signaling and promotes a reparative M2 phenotype [162]. Similarly, the flavonoid quercetin ameliorates ulcerative colitis by inhibiting cGAS-STING in intestinal macrophages, reducing M1 and enhancing M2 polarization to aid barrier repair [163]. Astragalus polysaccharide (APS) also alleviates kidney injury by suppressing macrophage-intrinsic cGAS-STING activation and M1 polarization [164]. Conversely, strategic pathway activation can be harnessed therapeutically. In GBM, tumor-associated macrophages (TAMs) are typically immunosuppressive and M2-like [165]. However, cGAMP-loaded nanocomplexes can reprogram these TAMs by activating their cGAS-STING pathway, driving a shift toward an immunostimulatory M1 phenotype to combat tumor growth [165] (Fig. 6).

In summary, a complex regulatory network exists wherein senescent cells, via their SASP, and the cGAS-STING pathway, both extrinsically and intrinsically, critically modulate macrophage polarization. This crosstalk represents a pivotal mechanism linking metabolic stress, DNA damage, and senescence to immune homeostasis. While the evidence for this interplay is compelling, the complete molecular circuitry remains to be fully delineated. Future research must systematically define the specific SASP factors that are the primary drivers of polarization, decipher the context-dependent outcomes of cGAS-STING signaling, and explore the integration with other immune pathways. A deeper understanding of these mechanisms will be instrumental in developing targeted immunomodulatory therapies.

### Non-canonical STING signaling in senescence: functional uncoupling and alternative activation mechanisms

The canonical view holds that cGAS-STING pathway activation follows a classical paradigm in which cytosolic DNA is recognized by cGAS, catalyzing the synthesis of the second messenger cGAMP. This subsequently induces STING translocation to the perinuclear region and phosphorylation, ultimately initiating type I interferon responses via the TBK1-IRF3 axis [51]. However, emerging studies have revealed that in aging and related pathological contexts, the signaling output of this pathway exhibits remarkable heterogeneity and functional plasticity. In progeria, senescent, and aging fibroblasts, despite the accumulation of cytosolic DNA, classical activation markers such as elevated cGAMP levels, STING phosphorylation, and its perinuclear translocation are not observed; instead, STING localizes to the endoplasmic reticulum, nuclear envelope, and chromatin regions [166]. Notably, these cells maintain SASP and interferon program expression in a cGAS- and STING-dependent manner, revealing a non-canonical STING signaling mode in senescence [166]. Consistently, during brain aging in mice, cGAS activity increases in the cortex without concurrent activation of downstream STING signaling, whereas in the hippocampus, STING protein accumulates but IRF3 phosphorylation is not enhanced [167]. This suggests a region-specific functional uncoupling of this pathway during the aging process.

At the mechanistic level, the discovery of multiple non-canonical STING activation pathways provides new perspectives for understanding aging-related inflammation. In the DNA damage response, keratinocytes and other cells can initiate cGAS-independent STING activation, a process mediated by the DNA-binding protein IFI16 in coordination with DNA damage response factors ATM and PARP-1, forming an alternative signaling complex containing p53 and TRAF6 [168]. TRAF6 catalyzes K63-linked polyubiquitination of STING, subsequently activating NF-κB and driving alternative gene expression programs. Furthermore, STING can directly interact with the endoplasmic reticulum kinase PERK, initiating the PERK-eIF2α signaling axis prior to TBK1-IRF3 activation, thereby regulating cap-dependent mRNA translation and establishing a translation program biased toward pro-inflammatory and pro-survival outcomes [169]. This pathway is evolutionarily primitive and plays a critical pathological role in cellular senescence and organ fibrosis. In a model of ischemia–reperfusion-induced liver injury, senescent macrophages exhibit enhanced STING-TBK1 signaling, which drives hyperactivation of the NLRP3 inflammasome, leading to massive secretion of pro-inflammatory cytokines such as IL-1β and IL-18. Inhibition of STING blocks this cascade and alleviates liver injury in aged mice, indicating that the STING-NLRP3 axis serves as a key regulatory node for the pro-inflammatory phenotype of senescent macrophages [170].

Therefore, the output mode of STING signaling undergoes profound remodeling during senescence, involving a transition from the canonical cGAS-cGAMP-STING-TBK1-IRF3 linear pathway to a complex regulatory network characterized by functional uncoupling and non-canonical alternative activation. This understanding not only reconciles seemingly contradictory experimental observations in previous studies but also holds important implications for targeted intervention strategies. In the context of aging, relying solely on the phosphorylation levels of canonical signaling molecules (e.g., p-TBK1, p-IRF3) as the sole criterion for pathway activation may be insufficient, and merely blocking these nodes may not fully suppress STING-driven pathological outputs. Future therapeutic strategies should focus on directly targeting the STING protein itself or its alternative activation nodes to precisely intercept its pro-aging pathological signaling output.

## The cGAS-STING pathway in aging-related multisystem diseases

The cGAS-STING innate immune pathway has emerged as a critical molecular hub linking genomic instability, mitochondrial dysfunction, and chronic inflammation in aging-related multisystem diseases. This pathway senses cytosolic DNA and becomes aberrantly activated during aging due to the accumulation of damage-associated molecular patterns (DAMPs), thereby driving degenerative pathologies across multiple organ systems, including cardiovascular, metabolic, skeletal, and ocular tissues [24, 26, 27, 171]. The following sections will systematically elaborate how the cGAS-STING signaling pathway promotes inflammaging and organ dysfunction through cell-type-specific mechanisms, with a particular emphasis on its emerging potential as a therapeutic target to delay aging-related pathological progression.

### Cardiovascular diseases

**Fig. 7:**
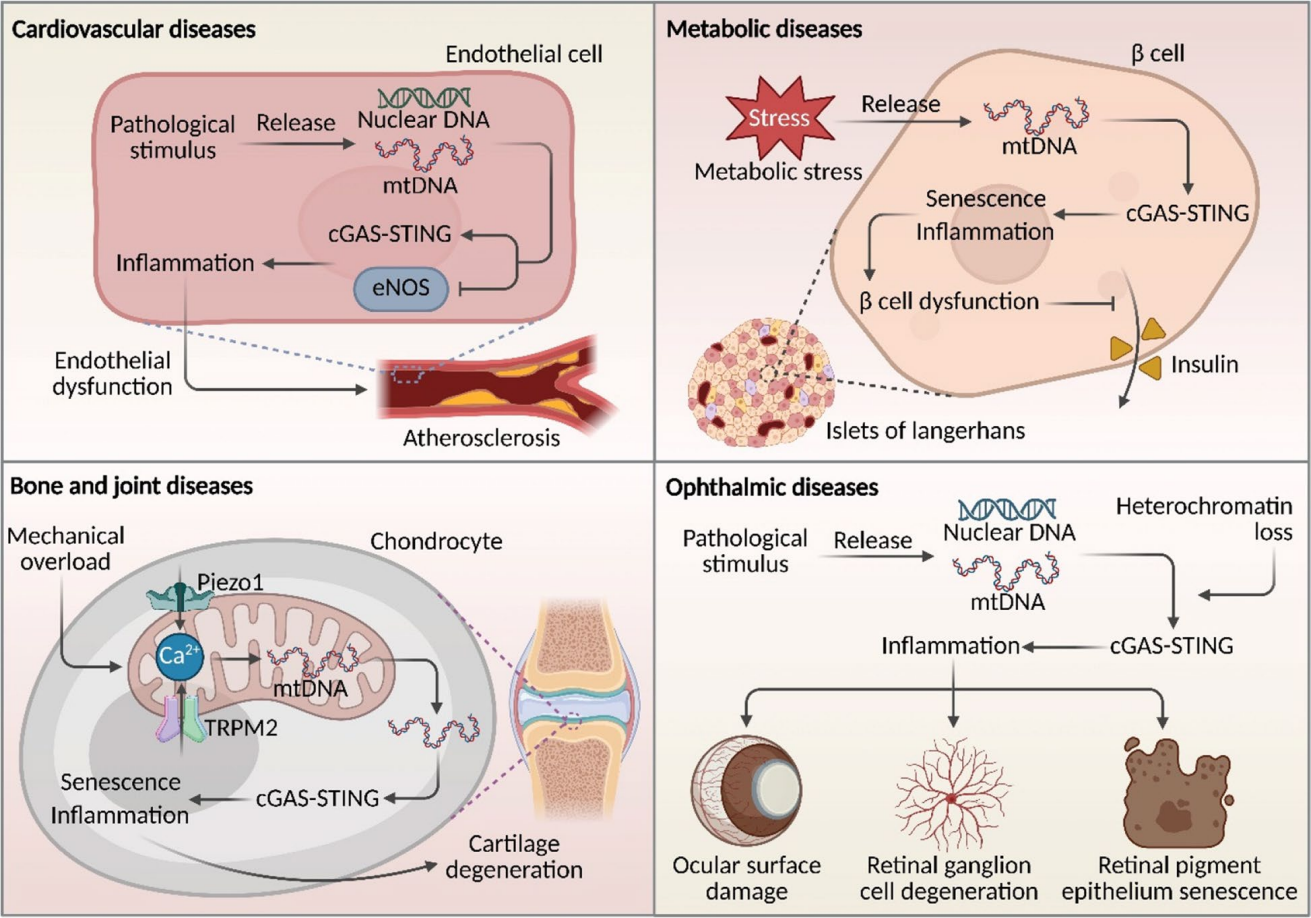
Pathogenic mechanisms of the cGAS-STING pathway in aging-related diseases. This illustration summarizes the central role of the cGAS-STING signaling pathway in the pathogenesis and progression of multiple aging-related diseases. Cardiovascular Diseases: Aging and related pathological stimuli induce the release of nuclear DNA and mtDNA from endothelial cells, activating the cGAS-STING pathway. This subsequently inhibits endothelial nitric oxide synthase (eNOS) function, exacerbating inflammatory responses and endothelial dysfunction, and ultimately promoting the development of atherosclerosis. Metabolic Diseases: Under metabolic stress, β-cells release mtDNA and activate cGAS-STING signaling, triggering cellular senescence and local inflammatory responses. This leads to β-cell dysfunction and impaired insulin secretion. Osteoarticular Disorders: Mechanical overload upregulates the expression of mechanosensitive ion channels Piezo1 and TRPM2 in chondrocytes, inducing Ca^2^⁺ influx and mitochondrial calcium overload. This subsequently causes mtDNA leakage into the cytoplasm, activating the cGAS-STING pathway, which drives cellular senescence, inflammation, and cartilage degeneration. Ophthalmic Diseases: Pathological stimuli promote the release of nuclear DNA and mtDNA in ocular cells, activating cGAS-STING signaling and initiating an inflammatory cascade. Heterochromatin loss further amplifies this pathway activity, resulting in ocular surface damage, retinal ganglion cell degeneration, and senescence of retinal pigment epithelial cells, thereby accelerating disease progression

The inexorable link between cardiovascular disease (CVD) and the aging process constitutes a cornerstone of geriatric medicine. Aging-associated cardiovascular decline is driven by a triad of key pathological processes: vascular endothelial dysfunction, cardiomyocyte senescence, and inflammaging. With aging, the accumulation of DNA damage and telomere dysfunction promotes a rise in senescent endothelial cells, which in turn exacerbate local oxidative stress and propagate inflammatory responses—for instance, via CCL4-mediated signaling—thereby significantly elevating overall CVD risk [172–174]. In parallel, senescence within the cardiomyocyte compartment contributes to aging-related cardiac pathology through telomere attrition, oxidative damage, and the sustained secretion of pro-inflammatory SASP factors. These changes collectively precipitate myocardial fibrosis, compromise systolic and diastolic function, and drive maladaptive cardiac remodeling [175–177]. Emerging evidence now implicates the innate immune system, and specifically the cGAS-STING signaling axis, as a pivotal orchestrator of cardiovascular aging [178–180]. This pathway, activated by the detection of cytosolic DNA, instigates potent inflammatory cascades and is increasingly regarded as a critical molecular bridge connecting fundamental aging mechanisms to cardiovascular pathogenesis (Fig. 7). Among the spectrum of CVDs, the roles of cGAS-STING signaling have been most extensively characterized in the contexts of atherosclerosis (AS) and heart failure (HF). The following sections will, therefore, critically examine the detailed mechanistic involvement and burgeoning therapeutic potential of targeting this pathway in AS and HF.

#### Atherosclerosis

AS is a chronic inflammatory disease of large and medium-sized arteries, pathologically characterized by lipid deposition, inflammatory cell infiltration, fibrous cap formation, and eventual plaque rupture [181–183]. While numerous risk factors contribute to its multifactorial etiology, the cGAS-STING pathway has emerged as a central innate immune regulator pivotal to several AS-related pathological processes [184–187]. For instance, cigarette smoke extract (CSE), a surrogate for the well-established AS risk factor of smoking, induces nuclear and mitochondrial DNA damage in human umbilical vein endothelial cells (HUVECs) [184]. The consequent accumulation of cytosolic DNA activates the cGAS-STING pathway, thereby driving chronic vascular inflammation, promoting foam cell formation, and contributing to plaque instability [184].

Senescence-associated endothelial dysfunction represents another key mechanism in AS pathogenesis. The expression of endothelial nitric oxide synthase (eNOS), a critical regulator of vascular tone via nitric oxide (NO) production, is inversely correlated with vascular aging [188, 189]. In D-galactose-induced senescent human aortic endothelial cells (HAECs), eNOS downregulation coincides with cGAS-STING pathway activation and increased IRF3 phosphorylation [24]. This signature is mirrored in aged human aortic tissues, which exhibit elevated levels of cGAS, STING, and phosphorylated IRF3 (p-IRF3) compared to younger counterparts [24]. Crucially, endothelial-specific STING deletion in murine models attenuates aging-related vascular impairments, including inflammation and mitochondrial dysfunction, thereby conferring protection against AS progression [66]. These findings collectively establish the cGAS-STING pathway as a major driver of senescence-induced endothelial dysfunction in AS. Beyond senescence, endothelial ferroptosis has recently been implicated in AS, likely through mechanisms involving endothelial injury and plaque development [190]. The binding of oxidized low-density lipoprotein (ox-LDL) to its primary endothelial receptor, lectin-like ox-LDL receptor-1 (LOX-1), activates the cGAS-STING pathway in HUVECs [191–193]. This activation upregulates nuclear receptor coactivator 4 (NCOA4), inducing ferritinophagy-dependent ferroptosis and exacerbating vascular endothelial injury and inflammation [193].

The role of cGAS-STING signaling extends beyond the endothelium to immune cells within the plaque microenvironment. Apolipoprotein E-deficient (ApoE⁻/⁻) mice, a canonical model of spontaneous hypercholesterolemia and AS, display heightened STING expression and DNA damage markers in aortic macrophages compared to wild-type mice [194, 195]. Genetic ablation of *Sting* in ApoE⁻/⁻ mice reduces atherosclerotic plaque burden in the aortic arch, diminishes macrophage accumulation, and downregulates pro-inflammatory gene expression in the aortic wall [195, 196]. Furthermore, VCAM-1-expressing macrophages in atherosclerotic plaques increase mtDNA synthesis under inflammatory stimuli, activating the cGAS-STING pathway and creating a self-perpetuating inflammatory cycle that exacerbates AS [197]. Amidst these pro-atherogenic mechanisms, endogenous protective pathways can counter disease progression. Aldehyde dehydrogenase 2 (ALDH2), for example, mitigates macrophage inflammatory responses and provides atheroprotection by suppressing cGAS-STING activation. This is achieved by reducing the interaction between ubiquitin-specific protease 14 (USP14) and cGAS [198].

In summary, the cGAS-STING pathway occupies a central position in AS pathogenesis by orchestrating innate immune responses, amplifying inflammatory cytokine production, and driving critical cellular events such as endothelial dysfunction and ferroptosis. Nevertheless, research on this pathway in AS remains nascent. Future investigations should prioritize elucidating its cell-type-specific molecular mechanisms, validating its components as viable clinical targets, and exploring its synergistic interactions with other inflammatory pathways in AS development.

#### Heart failure

HF represents a complex clinical syndrome arising from structural or functional cardiac impairment, culminating in inadequate cardiac output to meet metabolic demands and/or elevated ventricular filling pressures necessary to maintain perfusion [199–201]. The aging heart is particularly susceptible to HF, undergoing progressive cardiomyocyte loss, myocardial fibrosis, and mitochondrial dysfunction. These aging-related changes collectively impair diastolic function, reduce bioenergetic efficiency, and activate inflammaging, thereby establishing a substrate conducive to HF development [202–205]. Emerging research has now identified the cGAS-STING signaling pathway as a key contributor to HF pathophysiology, primarily through its roles in mediating mitochondrial dysfunction, sterile inflammation, fibrotic remodeling, and cardiomyocyte death [112, 159, 206].

Pressure overload (PO) is a fundamental pathogenic stimulus in HF, typically inducing concentric left ventricular hypertrophy that initially manifests as diastolic dysfunction (HF with preserved ejection fraction, HFpEF) and may progress to systolic impairment (HF with reduced ejection fraction, HFrEF) [207, 208]. Seminal work has established a critical role for cGAS in PO-induced HF, where it drives pathological cardiac remodeling and dysfunction [206]. In the transverse aortic constriction (TAC) mouse model—a well-established surrogate for PO—induction of inducible NO synthase (iNOS) promotes cytoplasmic mtDNA accumulation, leading to cGAS-STING activation in cardiomyocytes and subsequent sterile inflammation and cardiac dysfunction [159, 209, 210]. Furthermore, TAC-induced cardiac hypertrophy is associated with downregulation of PTEN-induced kinase 1 (PINK1), resulting in impaired mitophagy. This defect facilitates mtDNA leakage, which hyperactivates the cGAS-STING pathway and accelerates inflammatory-driven cardiac hypertrophy [211]. Importantly, pharmacological inhibition of cGAS-STING signaling attenuates PO-induced cardiac remodeling and interstitial fibrosis, potentially by modulating inflammatory responses and enhancing reparative M2 macrophage polarization [159].

The pathogenic relevance of cGAS-STING extends beyond PO to other etiologies of HF. Doxorubicin, a widely used chemotherapeutic agent, induces delayed cardiotoxicity and lethal HF via DNA damage-dependent activation of the cardiomyocyte cGAS-STING pathway and subsequent IFN-I signaling [212, 213]. This process involves doxorubicin-triggered mitochondrial permeability transition pore (mPTP) opening, mtDNA leakage, and the induction of myocardial senescence [179]. In diabetic cardiomyopathy (DCM), a diabetes-specific myocardial disorder that predisposes to HF, hyperglycemia impairs mitochondrial function via the SIRT1/AMPK/PGC-1α axis, prompting cardiomyocyte apoptosis and the release of mtDNA-containing extracellular vesicles [214, 215]. Fibroblasts internalize these vesicles, activating both TLR9 and cGAS-STING pathways to initiate pro-fibrotic signaling and maladaptive remodeling, highlighting a non-cardiomyocyte-centric mechanism [215]. Additionally, metabolic perturbations in the failing heart can exacerbate pathology. For instance, depletion of carnitine acetyltransferase (CRAT) in cardiomyocytes enhances cholesterol catabolism, inducing mtDNA stress and triggering a cGAS-STING-driven IFN-I response. This interferon signature subsequently upregulates AIM2 expression and inflammasome activation, perpetuating a cycle of chronic inflammation [180, 216].

In summary, diverse pathological insults in HF converge on a common mechanism: induction of mitochondrial damage and mPTP opening, leading to the cytosolic release of mtDNA. Acting as potent DAMPs, these mtDNA fragments activate the cGAS-STING pathway, driving the production of IFN-Is and pro-inflammatory cytokines that promote myocardial inflammation, fibrosis, and ultimate dysfunction. The high mitochondrial density and immense energy demands of cardiomyocytes may render them particularly vulnerable to this pathway. While these findings underscore the central role of cGAS-STING in HF, several critical questions remain. Future research must validate the universality of this mechanism in human HF patients, elucidate the potential contributions of other DAMPs, and rigorously assess the translational potential of therapeutic strategies targeting this axis, such as STING inhibitors.

### Metabolic diseases

The pathogenesis of metabolic disorders, including obesity, type 2 diabetes mellitus (T2DM), and non-alcoholic fatty liver disease (NAFLD), is intricately associated with the aging process. Advancing age is characterized by a progressive deterioration of metabolic homeostasis, hallmarked by the emergence of insulin resistance, mitochondrial dysfunction, and inflammaging [217–220]. These aging-related alterations collectively foster an environment conducive to ectopic lipid accumulation, elevated oxidative stress, and compromised cellular repair mechanisms, thereby driving a cascade of metabolic dysregulation and end-organ injury [221–223]. A growing body of evidence now indicates that these pathological cascades are frequently accompanied by the aberrant cytosolic accumulation of nucleic acids, such as mitochondrial and nuclear DNA fragments. These DAMPs potently activate the cGAS-STING signaling axis, instigating a persistent innate immune response and amplifying pro-inflammatory signaling networks (Fig. 7). Consequently, the cGAS-STING pathway is emerging as a pivotal molecular nexus that integrates metabolic stress, aging, and chronic inflammation.

#### Type 2 diabetes mellitus

T2DM is a complex metabolic disorder characterized by a dual pathogenesis of insulin resistance and the progressive functional decline of pancreatic β-cells, culminating in defective insulin secretion [224]. The aging process significantly exacerbates these core defects through mechanisms such as chronic inflammation, mitochondrial dysfunction, cellular senescence, and disrupted insulin signaling, thereby acting as a major contributor to T2DM pathogenesis [225–227]. Emerging research positions the aberrant activation of the cGAS-STING signaling pathway as a critical mechanistic link in this process [171, 228, 229].

Within pancreatic β-cells, metabolic stress can induce mitochondrial damage, leading to the cytosolic leakage of mtDNA. This, in turn, activates the cGAS-STING pathway, fueling a feed-forward loop of inflammatory signaling during cellular senescence that ultimately impairs β-cell function [171]. Beyond the pancreas, cGAS-STING activation mediates diabetes-associated pathology in extra-pancreatic tissues. For instance, in the diabetic brain, T2DM-related metabolic stress and lipotoxicity provoke mitochondrial dysfunction, mtDNA release, and subsequent cGAS-STING-driven neuroinflammation [228]. The therapeutic potential of targeting this axis is underscored by findings that the STING inhibitor C176 alleviates islet inflammation, senescence, and neuroinflammation in diabetic models, consequently improving β-cell function and glucose homeostasis [171, 228]. Adipose tissue dysfunction represents another critical pathophysiological process mediated by this pathway. Obesity, a primary risk factor for T2DM, exacerbates metabolic dysregulation by inducing cellular senescence and impairing white adipose tissue (WAT) function [230, 231]. Diet-induced obese (DIO) models exhibit pronounced inflammatory responses in WAT, which are pivotal for the development of obesity-associated insulin resistance [232]. Mechanistic insights reveal a functional interplay between the cGAS-STING pathway and STAT1/3 signaling in regulating senescence and inflammation in adipocytes, suggesting that in obesity-associated T2DM, cGAS-STING may drive adipose tissue dysfunction through crosstalk with other inflammatory pathways, thereby worsening insulin resistance [229].

The detrimental role of cGAS-STING signaling extends to the progression of T2DM complications. In diabetic nephropathy (T2DN), renal cGAS-STING activity escalates with disease progression [233]. Notably, intervention with nicotinamide riboside (NR) activates SIRT3, ameliorates mitochondrial fitness, and reduces mtDNA damage, thereby suppressing cGAS-STING activation and attenuating T2DN [234]. In diabetic retinopathy (DR), downregulation of PPARα in monocytes promotes mitochondrial damage and mtDNA release, activating cGAS-STING signaling and contributing to retinal leukostasis and microvascular injury [235]. Furthermore, T2DM can exacerbate comorbid conditions; for example, it aggravates Parkinson's disease (PD) pathology by inducing mtDNA-dependent cGAS-STING activation in microglia, which amplifies neuroinflammation and neuronal damage [236]. Similarly, T2DM exacerbates periodontitis, where cGAS-STING activation in human periodontal ligament stem cells (PDLSCs) drives cellular senescence and compromises regenerative capacity [237–240]. Promisingly, spermidine (SPD) can mitigate this effect by activating mitophagy to suppress STING signaling, thereby enhancing stem cell function and tissue repair [240]. Collectively, these findings establish the cGAS-STING pathway as a central mediator in the pathogenesis of diverse T2DM complications.

In summary, the cGAS-STING pathway serves as a critical molecular integrator in T2DM, translating metabolic and aging-related stress from mtDNA leakage into pervasive inflammation and cellular senescence, which drive β-cell failure, insulin resistance, and multi-organ damage. While therapeutic strategies such as STING inhibitors, NR, and SPD show considerable promise in preclinical models, several key challenges remain: (1) the tissue-specific regulatory mechanisms of cGAS-STING are poorly defined; (2) the current reliance on animal models necessitates validation in human clinical contexts; and (3) the crosstalk between cGAS-STING and other metabolic pathways (e.g., STAT1/3, PPARα) is inadequately explored. Future research should prioritize the validation of these mechanisms in human tissues, the development of tissue-targeted therapies, and the identification of non-invasive biomarkers to facilitate the precision treatment of T2DM and its complications.

#### Non-alcoholic fatty liver disease

NAFLD encompasses a spectrum of metabolic-linked liver pathologies, initiating with simple hepatocyte steatosis (defined by lipid accumulation in ≥ 5% of hepatocytes) [241] and potentially progressing through non-alcoholic steatohepatitis (NASH), fibrosis, cirrhosis, and ultimately, hepatocellular carcinoma (HCC) [242, 243]. As the most prevalent chronic liver disease globally, NAFLD pathogenesis is driven by a complex interplay of factors, including insulin resistance, dyslipidemia, oxidative stress, and chronic inflammation [244–247]. The aging process further accelerates NAFLD development and progression by exacerbating hepatic lipid accumulation, promoting SASP, and fostering inflammaging [248–250]. Mounting evidence now underscores a pivotal role for the aberrant activation of the innate immune cGAS-STING signaling pathway in this pathogenic cascade [25, 251]. A deeper mechanistic understanding of cGAS-STING regulation in NAFLD is therefore critical for unveiling novel therapeutic targets.

The cGAS-STING pathway exacerbates hepatic inflammation, fibrosis, and disease progression through several interconnected mechanisms. Central to this process is the genotoxic stress imposed by the lipotoxic NAFLD milieu. Under these conditions, proliferating hepatocytes experience nucleotide pool imbalances and replication stress, leading to persistent ATR/CHK1 activation and DNA damage [25]. Cytosolic DNA fragments are subsequently sensed by cGAS, which initiates STING-dependent IFN-I production, thereby establishing a chronic inflammatory microenvironment [25]. This mechanism positions the cGAS-STING axis as a critical molecular bridge between metabolic-induced genomic instability and innate immune activation, directly fueling the transition from NAFLD to NASH and fibrosis. Beyond endogenous DNA, exogenous nucleic acids from the gut microbiome also contribute to pathway activation. Under physiological conditions, hepatic Vsig4^+^ macrophages efficiently clear circulating microbiota-derived extracellular vesicles (mEVs) via complement C3-dependent mechanisms, preventing their accumulation in the liver [252, 253]. In NAFLD/NASH, however, the abundance of Vsig4^+^ macrophages is significantly reduced, leading to the aberrant accumulation of mEVs containing bacterial DNA in hepatocytes and hepatic stellate cells (HSCs) [254]. This microbial DNA potently activates the cGAS-STING pathway, inducing inflammatory responses in hepatocytes and driving HSC activation and collagen deposition, thereby directly promoting hepatic fibrogenesis [254].

Mitochondrial dysfunction represents another hallmark of NAFLD that is intricately linked to cGAS-STING signaling. In the NAFLD liver, dysregulation of selenoprotein W (SelW) promotes the nuclear translocation of pyruvate kinase M2 (PKM2), which activates the HIF-1α pathway [255]. This signaling cascade induces mitochondria-dependent apoptosis and ROS overproduction, culminating in mtDNA release. The concurrent ROS accumulation activates the NLRP3 inflammasome, triggering pyroptosis and the release of cell-free mtDNA. Upon internalization by macrophages, this mtDNA activates the cGAS-STING pathway, driving robust IFN-I responses and M1 polarization, which collectively exacerbate hepatic inflammation [255]. The discovery of this SelW–mtDNA–cGAS/STING axis reveals a novel mechanism whereby mitochondrial stress and innate immune dysregulation synergize to accelerate NAFLD progression. Notably, the role of cGAS-STING in NAFLD exhibits context-dependent complexity. While substantial evidence supports its predominantly pro-inflammatory and pro-fibrotic functions, emerging studies suggest a paradoxical, protective role under specific conditions. For instance, in high-fat, high-cholesterol, high-sucrose diet (HF-HC-HSD)-induced NASH models, genetic ablation of either *cGas* or *Sting* unexpectedly exacerbates hepatic steatosis, injury, and inflammation, potentially by compromising intestinal barrier integrity [256]. These seemingly contradictory findings highlight a dual regulatory role for the cGAS-STING pathway in NAFLD pathogenesis, the precise molecular underpinnings of which warrant further investigation.

In summary, the cGAS-STING pathway serves as a central hub in NAFLD/NASH progression, integrating signals from both endogenous (host-derived) and exogenous (microbiota-derived) nucleic acids to drive hepatic inflammation, fibrosis, and metabolic dysfunction. However, current research remains limited, particularly in reconciling the pathway's context-dependent dual roles. Future studies must prioritize the elucidation of spatiotemporal regulatory mechanisms governing cGAS-STING signaling across different disease stages and cell types. This refined understanding is a prerequisite for developing precise, effective therapeutic strategies that target this pathway for the treatment of NAFLD/NASH.

### Bone and joint diseases

Bone and joint diseases encompass a spectrum of complex pathologies driven by multifaceted etiologies, including mechanical stress imbalance, inflammatory mediator activation, and disruption of the joint microenvironment [257–259]. As quintessential aging-related disorders, their incidence and progression are strongly correlated with aging, which is considered a central risk factor [260]. The pathogenic cascade is primarily characterized by enhanced chondrocyte catabolism, dysregulated osteoclast activity, and the loss of subchondral bone homeostasis [261–263]. These conditions not only cause significant pain and functional impairment, severely reducing quality of life, but also pose a substantial socioeconomic burden. Recent research has illuminated the central role of the innate immune cGAS-STING pathway in bridging sterile inflammatory responses with joint tissue degeneration. By modulating inflammatory cascades and cellular senescence, this pathway is now recognized as a critical driver in the pathogenesis of osteoarthritis (OA) and rheumatoid arthritis (RA), positioning it as a promising therapeutic target [264, 265] (Fig. 7).

Accumulating evidence firmly establishes the pivotal role of cGAS-STING pathway activation in OA progression. Studies demonstrate that chondrocytes from OA models exhibit pronounced DNA damage alongside upregulated expression of cGAS and STING [26]. The genetic ablation of *Sting1* in mice subjected to the destabilization of the medial meniscus (DMM) model markedly attenuates OA pathology, reducing cartilage degradation and ameliorating subchondral bone sclerosis [26]. Conversely, intra-articular injection of the STING agonist cGAMP potently exacerbates OA-related damage. These genetic and pharmacological studies robustly validate the pathway's central role in OA pathogenesis. Building on this foundation, research has delineated how multiple pathological stimuli within the joint microenvironment converge to activate the cGAS-STING pathway. Mechanical overloading, a critical biomechanical driver of chondrocyte senescence, upregulates the mechanosensitive cation channel Piezo1 [266]. This promotes calcium influx, leading to mitochondrial calcium overload, subsequent mitochondrial dysfunction, and the cytosolic leakage of mtDNA, which ultimately triggers a cGAS-STING-dependent inflammatory response that accelerates cartilage breakdown [267]. Similarly, inflammatory stimuli upregulate the transient receptor potential melastatin 2 (TRPM2) channel [268]. By mediating calcium influx, TRPM2 induces mitochondrial damage and engages a pathogenic positive feedback loop involving cGAS-STING and NF-κB, thereby driving chondrocyte inflammation and degeneration [268]. Beyond calcium signaling, transferrin receptor-1 (TfR1)-mediated iron overload can also activate cGAS-STING via mitochondrial damage and mtDNA release, further exacerbating OA progression [269].

Once activated, the cGAS-STING pathway promotes joint degeneration through several effector mechanisms. In temporomandibular joint osteoarthritis (TMJOA), aberrant mechanical stress-induced mtDNA release activates cGAS-STING, which concurrently suppresses extracellular matrix (ECM) anabolism and promotes catabolism in chondrocytes, thereby accelerating cartilage degradation [270]. Furthermore, cGAS-STING activation induces metabolic reprogramming in chondrocytes, characterized by enhanced glycolysis, which further contributes to the degenerative process [270]. Notably, the regulatory purview of the cGAS-STING pathway extends beyond OA to inflammatory arthritides such as RA. DNA polymerase β (Pol β), a key enzyme in the BER pathway, has been identified as a crucial regulator in RA pathogenesis [271, 272]. Pol β deficiency results in the accumulation of unresolved DNA damage and the release of cytoplasmic dsDNA, which specifically activates the cGAS-STING–NF-κB innate immune axis [272]. This activation drives RA progression by promoting NLRP3 inflammasome-dependent macrophage pyroptosis (with consequent IL-1β/IL-18 secretion), exacerbating synovial inflammation, and enhancing osteoclast-mediated bone destruction [272]. Therapeutically, targeted inhibition of the cGAS-STING pathway holds significant clinical promise. For instance, the TBK1-specific inhibitor BX795 attenuates OA progression by concomitantly inhibiting both the cGAS-STING and TLR3-TRIF signaling pathways in chondrocytes, thereby reducing inflammation, mitigating cellular senescence, and preserving articular cartilage [273]. These findings provide a crucial mechanistic foundation for developing novel disease-modifying strategies aimed at this pathway.

In summary, the cGAS-STING pathway emerges as a pivotal regulator in degenerative joint diseases, functioning as a molecular sensor that detects both genomic and mitochondrial DNA damage to initiate persistent innate immune responses. These responses drive chondrocyte degeneration, suppress extracellular matrix synthesis, and exacerbate joint deterioration. By integrating diverse pathogenic stimuli—including mechanical stress and iron overload—this pathway establishes a self-amplifying cGAS-STING–NF-κB inflammatory circuit that functionally links mechanical damage, metabolic dysregulation, and progressive joint destruction, highlighting its potential as a therapeutic node for intervention.

### Ophthalmic diseases

Ophthalmic diseases comprise a spectrum of disorders that impair the structure and function of the visual system, frequently culminating in visual impairment or blindness. AMD, glaucoma, and cataracts exhibit particularly strong epidemiological links to the aging process [274–276]. Advancing age promotes disease susceptibility through morphological alterations in retinal pigment epithelial (RPE) cells, accumulation of endogenous DNA damage, and the establishment of inflammaging [277–279]. A growing body of evidence now implicates cellular senescence and genomic instability as key triggers of innate immune signaling, with the dysregulated activation of the cGAS-STING pathway emerging as a pivotal mechanism in the pathogenesis of these aging-related ocular conditions [280–282] (Fig. 7).

The cGAS-STING pathway is activated through disease-specific mechanisms across a range of ocular disorders, driving pathogenesis in a cell-type-dependent manner. In AMD, pathway activation occurs through interconnected mechanisms involving oxidative stress, genomic instability, and dysregulated iron metabolism [27, 283, 284]. Cytoplasmic DNA derived from damaged photoreceptors serves as a key activator of cGAS-STING in models of dry AMD and oxidative stress-induced retinal degeneration [27]. This inflammatory cascade is further amplified by aging-related progressive heterochromatin loss in the RPE and neural retina [283]. Furthermore, iron overload induces NCOA4-mediated ferritinophagy, releasing excessive free iron that causes mitochondrial impairment and robust cGAS-STING activation [284]. This iron-dependent mechanism significantly contributes to RPE senescence and the development of AMD-like pathology. Therapeutically, the iron chelator deferoxamine (DFO) can reverse this cascade, underscoring the iron–cGAS-STING axis as a promising therapeutic target for AMD [284]. In dry eye disease, hyperosmotic stress triggers mitochondrial oxidative damage, promoting mtDNA release into the cytoplasm through mPTP and subsequent cGAS-STING activation, which drives corneal epithelial inflammation and ocular surface damage [285, 286]. This mechanism has been validated in benzalkonium chloride (BAC)-induced mouse models, lacrimal gland excision models, and clinical patient samples [285].

The pathogenesis of glaucoma similarly involves aberrant cGAS-STING signaling. In glaucoma models, significant activation of the cGAS-STING–IRF3 axis in microglia drives detrimental microglia-macroglia interactions via IFN-I signaling, triggering pathogenic macroglial reactivity that results in retinal ganglion cell (RGC) degeneration and visual impairment [287]. Genetic ablation of *Sting1*, either globally or specifically in microglia, markedly attenuates glaucoma-associated RGC loss and visual dysfunction [287]. Moreover, the cGAS inhibitor A151 effectively mitigates neuroinflammation and preserves visual function by suppressing this pathway, highlighting its therapeutic potential [288]. In DR, breakdown of the inner blood-retinal barrier (iBRB) is a critical pathological event leading to vision loss [289]. Recent evidence reveals that cGAMP can activate the STING pathway in microglia via the P2RX7 receptor [290]. This mechanism not only contributes to iBRB structural breakdown but is also directly associated with progressive retinal neuronal degeneration [290]. These findings establish a novel link between metabolic dysregulation (cGAMP accumulation), neuroinflammation (microglial activation), and iBRB damage, providing fresh insights into DR pathogenesis.

In summary, the cGAS-STING pathway is activated in a cell-type– and stressor-specific manner across diverse ocular diseases, yet consistently promotes progression through sustained pro-inflammatory signaling. These multifaceted regulatory mechanisms offer potential biomarkers for the molecular stratification of ophthalmic disorders and establish a compelling rationale for developing novel immunomodulatory therapies targeting this pathway.

## Therapeutic strategies targeting the cGAS-STING pathway in aging-related diseases

Targeting the overactivation of the cGAS-STING pathway in aging-related diseases, multi-layered intervention strategies have been developed. These approaches not only include small molecule inhibitors directly targeting cGAS or STING proteins, but also involve indirect regulatory methods such as reducing DNA damage, clearing senescent cells, and blocking downstream signaling. Furthermore, cutting-edge technologies such as nanotechnology, novel biomaterials, and gene editing have provided new therapeutic avenues for the precise modulation of this pathway. The following sections will systematically elaborate on these therapeutic strategies targeting the cGAS-STING pathway and their application prospects in aging-related diseases (Table 1).

**Table 1:** Therapeutic strategies targeting the cGAS-STING pathway to inhibit aging-related diseases

Intervention strategy	Specific mechanism	Representative agent/molecule	Effects & applications (model)	References
cGAS inhibitors	Conventional small molecule inhibition	RU.521, PF-06928215, A151	Established preclinical research systems	[291–293]
	Structural optimization (2-azabicyclo[4.5]decane scaffold)	30d-S	35% oral bioavailability; exhibits significant anti-inflammatory efficacy in ALI model	[30]
	Allosteric inhibition (occupies both orthosteric and allosteric sites, stabilizes inactive conformation)	XL-3156, XL-3158	Inhibits LSPS, promotes LLPS, effectively attenuates cGAS-DNA interactions	[294]
	Metabolic regulation (Kla targets N-terminal lysine residues of cGAS)	L-lactate	Disrupts LLPS formation, impairs DNA recognition and cGAMP synthesis	[295]
STING inhibitors	Nitroalkylation of Cys88 and Cys91 residues	NO₂-FAs (Nitro-fatty acids)	Blocks palmitoylation, inhibits STING activity	[296]
	Structural optimization (enhanced electrophilicity and conformational restraint)	CP-36, CP-45	Demonstrates superior STING inhibitory activity; anti-inflammatory in animal models	[297]
	Blocks STING palmitoylation	Indole-urea derivatives (e.g., Compound 42)	Potent anti-inflammatory effects in Trex1^−/−^ mouse models with favorable pharmacokinetics	[298]
	Selectively targets Cys91 residue	GHN105 (Andromedane analogue)	Effective orally, significantly ameliorates pathological features in acute colitis models	[31]
	Binds to STING CTT, obstructs TBK1 recruitment	Carnosic Acid (CA)	Shows remarkable efficacy in autoinflammatory disease models	[299]

Intervention strategy	Specific mechanism	Representative agent/molecule	Effects & applications (model)	References
Reduce DNA damage	Inhibits MRE11 nuclease, prevents replication stress and micronucleus formation	Mirin	Suppresses cGAS-STING-dependent IFN responses	[32]
	Repairs telomeric damage, inhibits ATM/Chk1/p53 pathway	Tert overexpression	Blocks DNA damage signal transduction	[300]
	Modulates OGG1/FEN1 expression balance, reduces mtDNA-mediated signaling	6-Shogaol	Reduces mtDNA-mediated cGAS-STING signaling activation in the GI tract	[301]
	Reduces ROS generation, restores MMP	Fenofibrate	Inhibits mtDNA release, ameliorates corneal inflammation	[302]
	Upregulates Ambra1 to stabilize LONP1 and elevate PINK1 levels, enhances mitophagy	Urolithin A (UA)	Promotes clearance of damaged mitochondria, reduces mtDNA leakage	[303, 304]
	NAD^+^ precursor, prevents VDAC1-mediated mtDNA release under NAD^+^ depletion	Nicotinamide mononucleotide (NMN)	Effectively counteracts inflammation	[74, 305]
	Selective clearance of senescent cells to reduce DNA damage burden	ABT263, Dasatinib + Quercetin	Reduces oxidative stress, DNA damage, and SASP; restores impaired metabolism in obese models	[306, 307]
Block downstream signaling	Inhibits TBK1 activity through a dual phosphorylation mechanism	CDK4/6 (Inhibitors)	Suppresses TBK1 autophosphorylation, blocks STING signal transduction	[308]
	Binds to TBK1 kinase domain, disrupts trimeric complex formation	AKT1 (Kinase)	Leads to pathway inactivation	[309]
	Reduces TBK1 phosphorylation levels	BX795	Simultaneously suppresses cGAS-STING and TLR3-TRIF pathways	[273]
	Blocks nuclear translocation of phosphorylated IRF3	Indomethacin	Specifically suppresses type I interferon production	[310]
	Inhibits IκBα phosphorylation and degradation, prevents NF-κB nuclear translocation	BAY 11–7082	Reduces inflammatory cytokine release	[311, 312]
	Targets cytokine receptors (Biologics)	Tocilizumab (anti-IL-6R), Anakinra (anti-IL-1R)	Clinically available, but long half-lives and systemic immunosuppressive effects limit utility	[313, 314]
	Targets cytokines (Novel antagonists)	Novel mini-protein antagonists	High stability, precise targeting capability, and manufacturability make them promising alternatives	[314]
Nano-targeted delivery systems	Scavenges excess cerebral Cu^2^⁺ to inhibit ROS generation, activates nanozyme activity to catalyze NAD⁺ production and degrade H₂O₂	TP@PB-COF@NADH	Suppresses cGAS-STING pathway and neuroinflammation	[315]
	Binds damaged DNA, delivers inhibitor	CoAl-LDH (CAL) nanosheets + C176	Attenuates cGAS-STING signaling; significantly enhances anti-inflammatory effects in ALI models	[33]
	ROS-responsive, targeted delivery of cGAS inhibitor to inflamed colon tissues	SIMs (delivering RU.521)	Ameliorates intestinal pathology and restores epithelial barrier function	[316]
	Macrophage-specific targeting via "eat-me" signals, sustained release of STING inhibitor	Phosphatidylserine-modified nanoparticles (delivering C176)	Suppresses STING activation, promotes M2 polarization; shows long-term therapeutic potential for RNV treatment	[162]

Intervention strategy	Specific mechanism	Representative agent/molecule	Effects & applications (model)	References
Novel functional materials	Suppresses the ROS-cGAS-STING-NF-κB axis	tFNA (Tetrahedral framework nucleic acids)	Effectively alleviates inflammatory responses in dry eye disease	[317]
	Integrates ROS scavenging, mitochondrial repair, and cGAS-STING pathway inhibition	CeO₂-Y@ZIF-8@Gel	Significantly reduces endothelial cell damage while promoting an anti-inflammatory microenvironment	[318]
	Modulates the DNA damage-cGAS-STING pathway	FU/PVP-NAR nanoparticles	Suppresses renal tubular inflammation	[319]
Tissue engineering & genetic intervention	3D sustained-release system suppresses cGAS/STING signaling and ROS accumulation	HA-PDA NPs (delivering C176)	Effectively reduces ferroptosis while promoting cell differentiation; treats IVDD	[97]
	CRISPR-based genetic strategies targeting G4 structures in the STING promoter region	(CRISPR-based strategy)	Alleviates neuroinflammation in AD and restores microglial Aβ phagocytic capacity	[320]

### cGAS-STING inhibitors

The development of cGAS inhibitors exhibits a distinct dual-track approach, combining traditional and innovative strategies. In the field of conventional inhibitors, small-molecule compounds such as RU.521, PF-06928215, and A151 have established comprehensive preclinical research systems [291–293]. Meanwhile, significant breakthroughs have been made in the development of novel inhibitors based on new mechanisms and targets. In the context of structural optimization, the novel spiro[carbazole-3,3'-pyrrolidine] derivative 30d-S demonstrates enhanced cellular effects through its distinctive 2-azabicyclo[4.5]decane scaffold [30]. This compound exhibits 35% oral bioavailability and marked anti-inflammatory efficacy in an ALI model [30]. In terms of allosteric modulation, XL-3156 and XL-3158 can simultaneously occupy both the orthosteric and allosteric sites of cGAS. This dual-site binding mechanism stabilizes the activation loop in a closed inactive conformation, inhibits liquid–solid phase separation (LSPS) while promoting the transition to liquid–liquid phase separation (LLPS), thereby effectively attenuating cGAS-DNA interactions [294]. In the context of metabolic regulation, studies have revealed that L-lactate can target N-terminal lysine residues of cGAS through alanyl-tRNA synthetase 1/2 (AARS1/2)-mediated, ATP-dependent lactylation modification (Kla) [295]. This modification disrupts LLPS formation, consequently impairing DNA recognition and cGAMP synthesis.

Compared to cGAS inhibitors, STING inhibitors have broader application potential. The optimized STING inhibitors, derived from earlier compounds, exhibit superior performance. For instance, nitrated fatty acids (e.g., NO₂-FAs) inhibit STING activity by mediating nitroalkylation of Cys88 and Cys91 residues, thereby blocking its palmitoylation process [296]. Based on this mechanism, the structurally optimized 8-nitrostyrene derivatives CP-36 and CP-45 were designed with enhanced electrophilicity and conformational restraint properties [297]. These compounds demonstrate superior STING inhibitory activity and effectively ameliorate inflammation in animal models [297]. With advancing research, an increasing number of STING inhibitors featuring novel structures and unique mechanisms of action continue to emerge. For instance, indole-based urea derivatives (e.g., Compound 42) effectively block STING palmitoylation, demonstrating potent anti-inflammatory effects with favorable pharmacokinetic properties in Trex1^−/−^ mouse models [298]. The andromedane analogue GHN105 selectively targets STING's Cys91 residue, significantly ameliorating pathological features via oral administration in acute colitis models [31]. Additionally, carnosic acid (CA) binds to the STING C-terminal tail (CTT), obstructing TBK1 recruitment and consequently inhibiting IRF3 activation, showing remarkable efficacy in autoinflammatory disease models [299]. These advances not only deepen our understanding of the cGAS-STING regulatory mechanism but also lay the foundation for developing novel anti-inflammatory drugs.

### Indirect modulation strategies

While pharmacological agents directly inhibiting the cGAS-STING pathway have demonstrated promising therapeutic efficacy, certain limitations remain. Given that pathway activation and pathological effects depend on critical upstream and downstream mediators, indirect modulation strategies have consequently garnered significant attention, with several approaches already advancing to clinical investigation stages.

#### Reduce DNA damage

The cytoplasmic accumulation of endogenous DNA, stemming from genomic instability and mitochondrial dysfunction, constitutes a fundamental trigger for cGAS-STING pathway activation. Consequently, strategies that bolster genomic integrity or preserve mitochondrial function present promising avenues for suppressing aberrant innate immune signaling by mitigating the cytosolic presence of these DAMPs [300–302]. At the nuclear level, the replication stress and MN induced by oncogenic signaling are potent inducers of cGAS-STING activity. The MRE11 nuclease inhibitor Mirin effectively suppresses oncogene-induced, cGAS-STING-dependent interferon responses by preventing these DNA damage events [32]. Conversely, overexpression of telomerase reverse transcriptase (TERT) confers protection by not only repairing ROS-induced telomeric damage but also attenuating DNA damage signal propagation via inhibition of the ATM/Chk1/p53 pathway [300]. Furthermore, natural compounds offer therapeutic potential; for instance, 6-shogaol has been shown to significantly attenuate mtDNA-mediated cGAS-STING activation in the gastrointestinal tract by fine-tuning the expression balance between the base excision repair enzyme OGG1 and the endonuclease FEN1 [301].

mtDNA released into the cytosol is a major driver of cGAS activation. Current therapeutic strategies primarily aim to preserve mitochondrial fitness by targeting three key areas: stabilization of the mitochondrial membrane potential (MMP), enhancement of mitophagic clearance, and maintenance of NAD^+^ homeostasis [302, 305, 321]. For example, the lipid-lowering agent fenofibrate demonstrates dual efficacy by reducing ROS production and restoring MMP, thereby inhibiting mtDNA release and alleviating corneal inflammation [302]. The gut microbiome-derived metabolite urolithin A (UA) upregulates Ambra1, which stabilizes the LONP1 protease and elevates PINK1 levels, thereby promoting the selective clearance of damaged mitochondria and reducing mtDNA leakage [303, 304]. Additionally, nicotinamide mononucleotide (NMN), a key NAD^+^ precursor, counteracts inflammation by preventing VDAC1-mediated mtDNA release under conditions of NAD^+^ depletion [74, 305]. As previously discussed, the persistent DNA damage characteristic of cellular senescence perpetually activates the cGAS-STING pathway, driving SASP and propagating inflammation. Targeting this process at its source, senolytic drugs selectively eliminate senescent cells to reduce the overall burden of DNA damage, thereby attenuating chronic cGAS-STING signaling. The senolytic compound ABT263, for instance, produces significant therapeutic benefits by specifically clearing senescent bone cells, which markedly reduces local oxidative stress, DNA damage, and SASP factor secretion [306]. Furthermore, the combination of dasatinib and quercetin not only depletes the senescent cell population but also restores impaired fatty acid metabolism, oxidative phosphorylation, and the expression of DNA repair-related genes in models of obesity [307]. Collectively, these findings delineate a multi-tiered interventional strategy for treating chronic inflammatory aging-related diseases, focusing on upstream preservation of genomic and mitochondrial integrity to preemptively restrain the cGAS-STING pathway.

#### Block downstream signaling

Hyperactivation of the cGAS-STING pathway is strongly implicated in chronic inflammation and aging-related pathologies. Downstream signaling from this pathway bifurcates upon TBK1 phosphorylation, activating two principal arms: the IRF3-mediated IFN-I response and the NF-κB-driven inflammatory cascade. In recent years, regulatory strategies targeting these downstream nodes have emerged as a pivotal research focus for mitigating inflammatory diseases. The phosphorylation status of TBK1 has been identified as a central regulatory hub governing STING signal transduction [273, 308]. For instance, CDK4/6 kinases inhibit TBK1 through a dual mechanism: (i) direct phosphorylation of TBK1 at Serine 527 inactivates the kinase, while (ii) phosphorylation of RB1 (at S249/T252) promotes RB1-TBK1 complex formation, thereby suppressing TBK1 autophosphorylation at the critical S172 residue and ultimately blocking STING signaling [308]. Similarly, AKT1 hyperactivation disrupts the formation of the TBK1/STING/IRF3 trimeric complex via direct binding to the TBK1 kinase domain, leading to pathway inactivation [309]. Furthermore, small-molecule inhibitors such as BX795 can concurrently suppress both cGAS-STING and TLR3-TRIF pathway activation by reducing TBK1 phosphorylation levels [273].

Regarding the IRF3 branch, the drug indomethacin specifically attenuates IFN-I production by impeding the nuclear translocation of phosphorylated IRF3 [310]. For the NF-κB signaling axis, inhibitors including BAY 11–7082 effectively prevent NF-κB nuclear translocation by inhibiting IκBα phosphorylation and subsequent degradation, consequently reducing the release of inflammatory cytokines [311, 312]. Notably, chromosomal instability (CIN) can cooperatively activate the cGAS-STING-mediated IL-6–STAT3 signaling axis via the non-canonical NF-κB pathway (RELB-dependent), highlighting the necessity of targeting broader cytokine networks [313]. Although biologics like tocilizumab (anti-IL-6R) and anakinra (anti-IL-1R) are clinically available, their long half-lives and propensity for systemic immunosuppression limit their utility in managing acute or localized inflammation [313, 314]. Consequently, novel mini-protein antagonists have surfaced as promising alternatives due to their high stability, precise targeting capability, and favorable manufacturability [314]. In conclusion, multi-tiered suppression of cGAS-STING-mediated inflammatory output can be achieved by targeting TBK1 phosphorylation, IRF3/NF-κB nuclear translocation, or downstream cytokine signaling. Future investigations should prioritize refining drug specificity and delivery strategies to optimally balance anti-inflammatory efficacy with the preservation of immune homeostasis.

### Emerging technologies

The past decade has witnessed groundbreaking advances in intervention strategies designed to counteract the excessive activation of the cGAS-STING signaling pathway. In contrast to conventional inhibitors, emerging therapeutic technologies offer novel avenues for treating aging-related diseases, distinguished by their precise targeting, dynamic regulatability, and multi-faceted therapeutic potential. Notable progress has been made in the development of nanomaterial-based delivery systems, which leverage unique biocompatibility and multifunctionality to achieve targeted immunomodulation. For instance, an endogenous copper ion–responsive covalent organic framework (TP@PB-COF@NADH) suppresses cGAS-STING–driven neuroinflammation by scavenging excess cerebral Cu^2^⁺ to inhibit ROS generation, while concurrently activating nanozyme activity to catalyze NAD⁺ production and degrade H₂O₂ [315]. Inhaled CoAl-LDH (CAL) nanosheets attenuate cGAS-STING signaling through damaged DNA binding, and their combination with the inhibitor C176 significantly enhances anti-inflammatory efficacy in ALI models [33]. Similarly, ROS-responsive nanomicelles (SIMs) enable targeted delivery of the cGAS inhibitor RU.521 to inflamed colon tissues, ameliorating intestinal pathology and restoring epithelial barrier integrity [316]. Furthermore, phosphatidylserine-modified dendritic mesoporous silica nanoparticles exploit “eat-me” signals for macrophage-specific targeting, enabling sustained release of C176 to suppress STING activation and promote M2 polarization, demonstrating single-dose long-term therapeutic potential in retinal neovascularization [162].

In parallel, novel functional biomaterials are expanding the toolbox for cGAS-STING pathway modulation. Tetrahedral framework nucleic acids (tFNA) effectively alleviate inflammatory responses in dry eye disease by suppressing the ROS–cGAS–STING–NF-κB axis [317]. Multifunctional composites such as CeO₂-Y@ZIF-8@Gel integrate ROS scavenging, mitochondrial repair, and cGAS-STING pathway inhibition, significantly reducing endothelial cell damage while fostering an anti-inflammatory microenvironment [318]. Natural product–based carriers, including FU/PVP-NAR nanoparticles, exhibit therapeutic potential by modulating the DNA damage–cGAS–STING pathway to suppress renal tubular inflammation [319]. The field of tissue engineering and genetic intervention is also yielding innovative approaches with substantial translational promise. For example, in intervertebral disc degeneration (IVDD), a 3D sustained-release system based on methacrylated hyaluronic acid and C176-loaded polydopamine nanoparticles concurrently reduces ferroptosis and promotes cell differentiation via continuous suppression of cGAS-STING signaling and ROS accumulation [97]. CRISPR-based genetic strategies targeting G-quadruplex (G4) structures within the STING promoter not only alleviate neuroinflammation in Alzheimer’s disease but also restore microglial Aβ phagocytic capacity [320]. Collectively, these next-generation technologies establish a new paradigm for the precise treatment of cGAS-STING–related disorders through multi-scale intervention (molecular/cellular levels), dynamic responsiveness (e.g., to ROS), and synergistic mechanisms (e.g., nanozyme–drug combinations). Future research should prioritize the optimization of targeting efficiency, long-term biosafety, and viable clinical translation pathways to catalyze transformative breakthroughs in this rapidly evolving field.

## Current challenges and where is the filed going

The cGAS-STING pathway not only drives aging-related pathologies but also serves as a critical regulator of physiological homeostasis. In tumor immune surveillance, this pathway recognizes tumor-derived DNA and induces STING-dependent type I interferon (e.g., IFN-β) production, thereby initiating CD8⁺ T cell-mediated antitumor immunity [322]. Intrinsic cGAS-STING activation in T cells also amplifies interferon signals in an autocrine manner, promoting the differentiation of stem-like CD8⁺ T cells with durable memory potential, thereby supporting adoptive T cell therapies [323]. In host defense, as a key cytosolic DNA sensor, the pathway rapidly detects nucleic acids from DNA viruses (e.g., herpesviruses) and intracellular bacteria (e.g., *Leptospira interrogans*), inducing robust type I interferon responses and establishing a broad antimicrobial defense [324, 325]. Furthermore, this pathway participates in tissue repair and cellular quality control. In skin injury, localized, moderate cGAS-STING activation coordinates immune cell recruitment and epithelial regeneration by inducing type I interferons and the chemokine CXCL10, thereby accelerating wound healing [326]. Recent studies have also revealed that STING activation induces nuclear translocation of transcription factor EB, enhancing lysosomal biogenesis and autophagic flux to facilitate the clearance of aberrant cytosolic DNA [327]. Collectively, these functions illustrate the context-dependent nature of cGAS-STING signaling. Moderate, physiological activation serves as a guardian of immune surveillance, host defense, and tissue repair, whereas its chronic overactivation drives inflammaging and related diseases.

Given its central role in the aforementioned physiological processes, systemic or long-term inhibition of the cGAS-STING pathway may pose multiple safety concerns. The primary risk is the potential induction of a systemic immunosuppressive state. As a critical hub for recognizing viral and intracellular bacterial DNA and initiating type I interferon responses, broad inhibition of this pathway would significantly compromise host defense, increasing susceptibility to infections [328]. More critically, this pathway is essential for initiating and maintaining adaptive antitumor T cell immunity; long-term or non-specific inhibition may impair endogenous tumor immune surveillance, potentially undermining the efficacy of existing immunotherapies and even creating conditions conducive to tumor development or recurrence [323, 329]. Furthermore, the cGAS-STING pathway plays a role in maintaining genomic integrity in human cells, and its non-selective inhibition may disrupt genomic homeostasis, thereby posing a potential risk of triggering autoimmune responses [60].

Currently, drug development targeting the cGAS-STING pathway is progressing rapidly, yet no agents have been approved for aging or aging-related indications. Research and development in this field primarily focus on two directions, namely the development of pathway agonists to enhance antitumor immunity in oncology and the exploration of inhibitors to suppress excessive inflammation in autoimmune diseases [28, 29]. Preclinical studies in aged or diseased animal models have demonstrated that modulating this pathway can improve aging-related phenotypes and alleviate various pathological conditions, with promising results reported [31, 307]. However, to date, no inhibitor has entered clinical trials with "delaying aging" or "treating aging-related syndromes" as its primary indication. The inherent double-edged sword nature of the cGAS-STING pathway makes inhibitor development particularly challenging, it requires effectively suppressing aberrant activation while avoiding excessive inhibition that could compromise immune function. This reality underscores the complexity and scientific challenges involved in translating pathway modulation strategies to chronic, systemic aging processes.

To achieve an optimal balance between therapeutic efficacy and safety, and to facilitate the clinical translation of this target, interventions targeting the cGAS-STING pathway must evolve toward greater precision and context-dependence. The current core technological challenges include the high heterogeneity of senescent cells and the lack of specific surface markers [330, 331]. This makes the design of precision targeted delivery systems particularly difficult. Concurrently, ensuring the efficient enrichment of therapeutic agents in target cell populations while minimizing off-target effects on normal cells, especially immune cells, presents another major challenge. Addressing these challenges requires multidimensional innovative strategies. These include developing intelligent delivery systems responsive to specific cell types or microenvironments, such as prodrug designs activated only within target cells; utilizing senescence-associated biomarker profiles (e.g., surface proteomes, secretomes, and metabolic features) for patient stratification and therapeutic guidance; and exploring spatiotemporally controllable intervention modalities, such as local administration systems and drug release mechanisms activated by specific pathological microenvironmental signals (e.g., enzymatic activity, pH changes). The integrated development of these precision medicine strategies holds promise for effectively modulating the pathological activity of the cGAS-STING pathway while maximally preserving its physiological protective functions, thereby opening new avenues for the treatment of aging and aging-related diseases.

## Conclusions

**Fig. 8:**
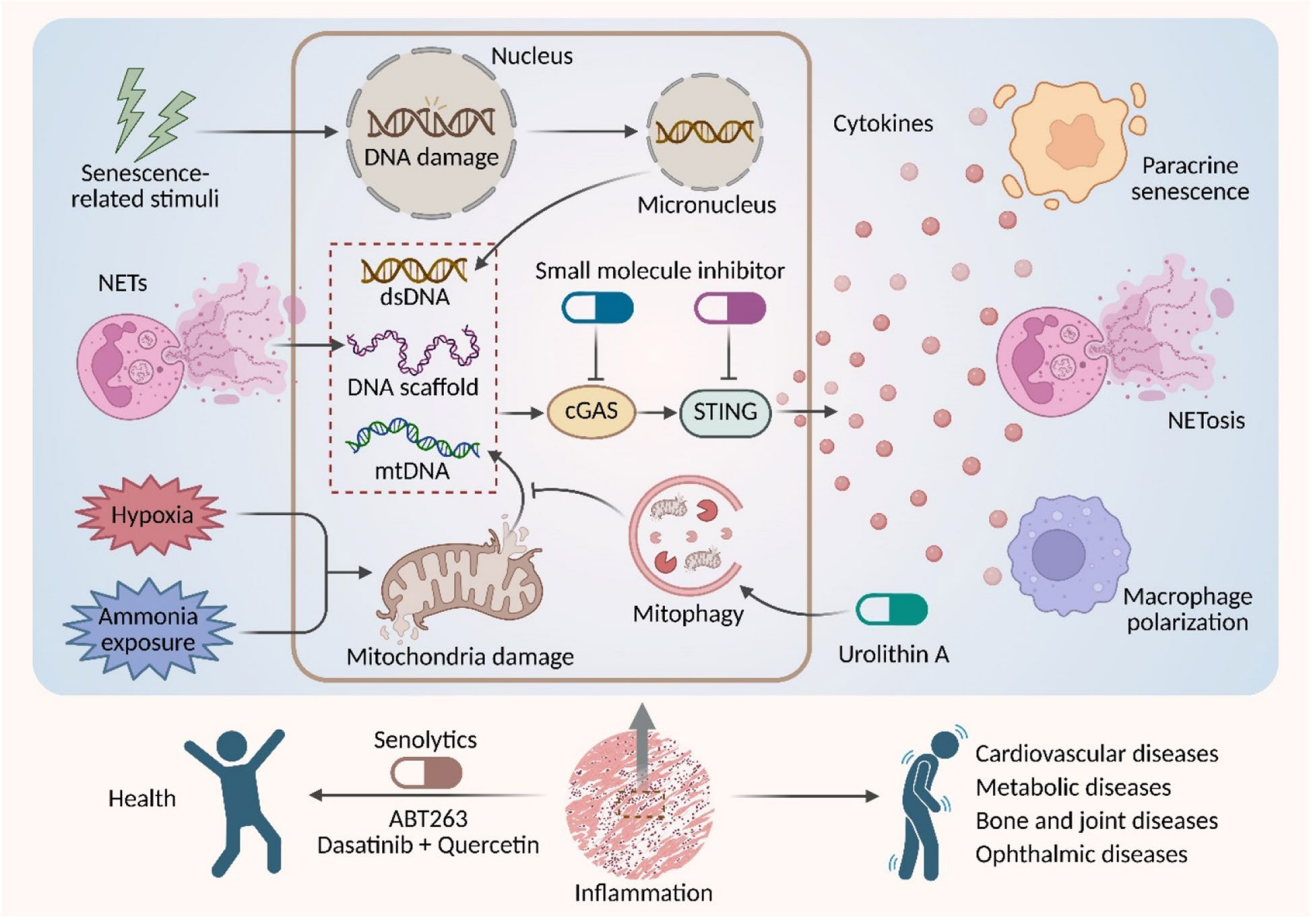
cGAS-STING integrates stress to drive senescence and inflammation. The cGAS-STING pathway integrates various stress signals (including DNA damage, hypoxia, ammonia-induced cell death, and NETs, among others) and serves as a central pathway driving cellular senescence and inflammatory responses. By recognizing accumulated cytoplasmic damaged DNA (e.g., mtDNA, nuclear DNA fragments, and NET-derived DNA), this pathway not only induces cell-autonomous senescence but also promotes the secretion of cytokines. These cytokines propagate the senescent phenotype to neighboring cells through paracrine effects, while simultaneously exacerbating inflammatory responses by promoting NETosis and macrophage polarization. This creates a self-amplifying positive feedback loop, playing a critical role in the development and progression of aging-related diseases. The figure also outlines potential therapeutic strategies aimed at disrupting this cycle, including direct cGAS/STING inhibitors, compounds such as Urolithin A that enhance mitophagy to limit mtDNA release, and senolytic agents that selectively eliminate senescent cells

As a core DNA-sensing mechanism of the innate immune system, the cGAS-STING pathway serves as a hub connecting diverse aging-related stresses with sterile inflammation. The triggers for this pathway are varied. They include genotoxic stress-induced DNA damage, hypoxia caused by energy metabolic imbalance, ammonia toxicity from metabolic waste accumulation, and NETs formed upon immune cell hyperactivation. Despite their diversity, these stress signals converge through a common endpoint, namely the disruption of nuclear or mitochondrial membrane integrity. This disruption leads to aberrant leakage and cytosolic accumulation of endogenous DNA, including nuclear DNA fragments, mtDNA, and NET-derived DNA. Once present in the cytosol, these normally sequestered "self" DNAs are recognized by cGAS as danger signals, thereby uniformly activating STING-dependent downstream inflammatory and senescence programs. Notably, activation of the cGAS-STING pathway not only directly induces cell-autonomous senescence but also establishes a self-perpetuating positive feedback loop by driving the release of SASP factors. The SASP propagates the senescent phenotype to neighboring cells through paracrine mechanisms; its specific components (e.g., IL-8, TNF-α) further promote NETosis and induce macrophage polarization toward a pro-inflammatory M1 phenotype, thereby perpetuating and amplifying inflammatory responses within the local microenvironment. This multi-layered regulatory network demonstrates significant pathogenic roles across various aging-related diseases. Compelling evidence demonstrates that cGAS-STING activation accelerates disease progression by promoting inflammatory cytokine production across diverse stress conditions, cell types, and disease contexts. Consequently, targeted inhibition of the cGAS-STING pathway represents a promising therapeutic strategy. Beyond direct inhibitors, we highlight innovative indirect modulation approaches and emerging technologies that show considerable translational potential **(**Fig. 8).

Despite significant advances, several critical knowledge gaps remain to be elucidated. First, whether distinct stressor-specific molecular signatures exist for cGAS-STING activation remains unclear. For instance, while hypoxia primarily activates the pathway through HIF-1α-dependent mitochondrial damage, ammonia-induced cell death involves unique lysosome-mitochondria crosstalk—whether these differences can be exploited for specific inhibitor development requires further investigation. Second, the spatiotemporal regulation of cGAS-STING in paracrine senescence remains poorly understood, particularly regarding how SASP factors selectively activate STING signaling in neighboring cells. Moreover, the pathogenic significance of the NET-cGAS-STING positive feedback loop in age-related chronic inflammation, along with the therapeutic advantage of combinatorial strategies (e.g., simultaneous inhibition of NETosis and STING signaling), requires systematic validation. Addressing these knowledge gaps represents a critical research priority. Future investigations should integrate cutting-edge technologies including single-cell spatiotemporal omics, organoid models, and AI-based predictive approaches to develop biomarkers distinguishing physiological (e.g., anti-infection) from pathological (e.g., senescence-associated) STING activation and design microenvironment-responsive smart delivery systems for precise targeting of specific cell populations (e.g., senescent cells or hyperactivated macrophages).

## Data Availability

No datasets were generated or analysed during the current study.
